# Comparative transcriptome analysis revealed resistance differences of Cavendish bananas to *Fusarium oxysporum* f.sp. *cubense* race1 and race4

**DOI:** 10.1186/s12863-020-00926-3

**Published:** 2020-11-11

**Authors:** Honghong Dong, Yiting Ye, Yongyi Guo, Huaping Li

**Affiliations:** Guangdong Province Key Laboratory of Microbial Signals and Disease Control, College of Agriculture, South China Agricultural University, Guangzhou, 510642 China

**Keywords:** Brazilian, *Fusarium oxysporum* f. sp. *cubense*, RNA-seq, Interaction, Resistance differences

## Abstract

**Background:**

Banana Fusarium wilt is a devastating disease of bananas caused by *Fusarium oxysporum* f. sp. *cubense* (Foc) and is a serious threat to the global banana industry. Knowledge of the pathogenic molecular mechanism and interaction between the host and Foc is limited.

**Results:**

In this study, we confirmed the changes of gene expression and pathways in the Cavendish banana variety ‘Brazilian’ during early infection with Foc1 and Foc4 by comparative transcriptomics analysis. 1862 and 226 differentially expressed genes (DEGs) were identified in ‘Brazilian’ roots at 48 h after inoculation with Foc1 and Foc4, respectively. After Foc1 infection, lignin and flavonoid synthesis pathways were enriched. Glucosinolates, alkaloid-like compounds and terpenoids were accumulated. Numerous hormonal- and receptor-like kinase (RLK) related genes were differentially expressed. However, after Foc4 infection, the changes in these pathways and gene expression were almost unaffected or weakly affected. Furthermore, the DEGs involved in biological stress-related pathways also significantly differed after infection within two Foc races. The DEGs participating in phenylpropanoid metabolism and cell wall modification were also differentially expressed. By measuring the expression patterns of genes associated with disease defense, we found that five genes that can cause hypersensitive cell death were up-regulated after Foc1 infection. Therefore, the immune responses of the plant may occur at this stage of infection.

**Conclusion:**

Results of this study contribute to the elucidation of the interaction between banana plants and Foc and to the development of measures to prevent banana Fusarium wilt.

**Supplementary Information:**

**Supplementary information** accompanies this paper at 10.1186/s12863-020-00926-3.

## Background

Banana (*Musa* spp.) is among the paramount fruit corps worldwide due to its high nutritive value [1]. Banana Fusarium wilt, which is caused by *Fusarium oxysporum* f. sp. *cubense* (Foc), is one of the most destructive plant diseases that affects banana production worldwide. It has led to considerable production losses since it was first discovered in Australia in 1876 [2]. Foc has been divided into three physiological races, race1 (Foc1), race2 (Foc2), and race4 (Foc4), on the basis of host type and pathogenicity. These races attack different banana cultivars. Foc1 and Foc4 are the most important races owing to their extensive distribution in South China and considerable yield loss.

The Foc1 resistant cultivar ‘Cavendish’ (AAA) subgroup has dominated banana exports since the 1960s, thereby becoming the major commercial variety in the world [3]. The Cavendish banana variety ‘Brazilian’ can be infected by Foc1 and Foc4, but it is resistant to Foc1 and susceptible to Foc4 [4–7]. Many researchers have focused on discovering the reason for the difference between the resistance to Foc1 and Foc4. ‘Brazilian’ adopts several strategies to cope with Foc infection [8]. Resistant varieties can prevent pathogen colonization in banana root by inhibiting Foc spore germination [9]. The reconstitution of the cell walls of banana roots after Foc challenge involves changes in the methylesterification of pectin and the distribution and abundance of extensins and arabinogalactan proteins [6, 10, 11]. These dynamic changes have a dramatic effect on banana resistance to Foc. In addition, genes related to plant hormone signaling, antioxidant defense, and classical defense have been suggested as candidates for studying banana resistance against Foc [12, 13]. DNA methylation may also contribute to banana resistance [14]. Li et al. [15] considered whether the further expansion of Foc spores to the rhizome tissue is a reason for the differences between the resistance of ‘Brazilian’ to the two Foc races. Fan et al. [6] showed that the difference in pectin methylesterase activity induced by both Foc races contributes to the resistance difference of ‘Brazilian’. Fan et al. [5] also indicated that the contents of oligogalacturonide in plants after Foc infection are related to this difference. Hyphal enrichment, infection rate, expansion range, and fusaric acid accumulation after Foc infection in plant tissues contribute to the differences in resistance [4]. These results contribute to the understanding of the resistance mechanism in banana.

High-throughput sequencing is extensively utilized to study the resistance mechanisms of plants, the interaction between hosts and pathogens, and the identification of promising resistant targets [16]. Some studies have applied these techniques to illustrate the defense mechanism of Cavendish banana response to Foc infection. For example, Wang et al. [17] revealed the changes in the transcriptome level of ‘Brazilian’ after Foc4 infection using RNA-Seq analysis. Li et al. [18], Bai et al. [19] and Niu et al. [16] used tissue-cultured plants and young micropropagated seedlings to analyze the transcriptomes of resistant and susceptible Cavendish cultivars after infection with Foc4, respectively. Wang et al. [20] compared the root transcriptomes of ‘Formosana’ and ‘Brazilian’ plantlets infested with Foc4 under field conditions. Similarly, Sun et al. [21] studied the comparative transcriptome of Foc4 infected with the resistant variety ‘Guijiao 9’ and the susceptible variety ‘Brazilian’. In addition, Zhang et al. [22] subjected the rhizome samples of the resistant variety ‘Pahang’ and the susceptible variety ‘Williams’ to transcriptome analysis after Foc4 infection. The above studies were based on the comparative transcriptome analysis of susceptible varieties infected with Foc4 alone or simultaneous infection with disease-resistant and susceptible varieties. However, a comparison of the infection of a Cavendish cultivar, such as ‘Brazilian’, with pathogenic Foc4 and nonpathogenic Foc1 will provide a highly efficient approach to determine resistance mechanisms. Recently, only Li et al. [23] used a pooled sample of the different tissues of ‘Brazilian’ for comparative gene expression analysis, with a focus on improving annotation of banana genomes, and found over 842 genes are not annotated by the *Musa* genome project. Nevertheless, detailed information regarding to the differences in gene expression and the affected pathways in ‘Brazilian’ plants after infection with Foc1 and Foc4 has not yet been reported. Our previous quantitative proteomic analysis attempted to reveal the molecular mechanism on ‘Brazilian’ root infection by Foc1 and Foc4. A series of plant resistance-related proteins are differentially accumulated after infection with both Foc races, indicating that the resistance of ‘Brazilian’ against two races differs [24]. Although several vital clues have been obtained from intensive proteomic research on ‘Brazilian’ roots after inoculation with Foc, a comprehensive analysis of global transcriptome responses to the different pathogenicity of two Foc races in ‘Brazilian’ has not been performed.

In this study, we performed a comparative transcriptome analysis to identify differentially expressed genes (DEGs) and compared different defense responses of ‘Brazilian’ after inoculation with Foc1, Foc4, and mock control. Our main objective was to investigate whether different defense responses are involved in the regulating of resistance differences of ‘Brazilian’ against Foc1 and Foc4. Moreover, a physiological and biochemical method was used to determine the changes of secondary metabolites in ‘Brazilian’ after Foc infection. We revealed that the differential expression of specific receptor-like kinases (RLKs), transcription factors (TFs), secondary metabolites, and plant hormone-related genes played a substantial role in the ‘Brazilian’ resistance difference to Foc1 and Foc4. Our results emphasized the different transcriptional reactions of ‘Brazilian’ plants after infection with two different races of Foc.

## Results

### Determining the time-points for harvesting the samples and comparative transcriptome analysis

At 48 h after inoculation (hai), a clear difference between the infection process of Foc1 and Foc4 was observed. Most of the spores of Foc had germinated and developed into hyphae, but part of the hyphae of Foc4 rather than Foc1 began to grow along with the gaps of epidermal cells, and the pathogen began to invade the root outer epidermal cells (Additional file 2: Fig. S1). In a previous work, we selected the samples at 48 hai for comparative proteomic analysis [24]. In this study, we also selected samples at 48 hai to investigate the differential transcriptomics of ‘Brazilian’ roots in response to infection with both Foc races.

A total of 60,560,402, 71,856,014, 48,606,378, 51,109,106, 54,540,798, 46,434,092, 51,623,832, 62,057,528, and 45,487,338 clean reads were generated from the nine libraries (CK-1, CK-2, CK-3, Foc1-1, Foc1-2, Foc1-3, Foc4-1, Foc4-2, and Foc4-3). Among the data of the nine clean reads libraries, 83.74 to 87.90% of the clean reads were successfully mapped to the reference genome (Table 1).

**Table 1:** RNA-seq data quality of nine sample from ‘Brazilian’ root

Sample	Clean reads	Error (%)	Q20	Q30	Mapping ratio (%)
CK-1	30,280,201	0.03	95.86	89.83	83.95
CK-2	35,928,007	0.03	98.04	94.26	85.68
CK-3	24,303,189	0.02	98.20	94.64	87.44
Foc1-1	25,554,553	0.02	98.06	94.27	87.90
Foc1-2	27,270,399	0.03	98.00	94.19	87.71
Foc1-3	23,217,046	0.03	97.50	92.94	84.36
Foc4-1	25,811,916	0.02	98.05	94.29	87.33
Foc4-2	31,028,764	0.02	98.24	94.79	86.16
Foc4-3	22,743,669	0.03	95.83	89.76	83.74

### Analysis and verification of DEGs

**Fig. 1:**
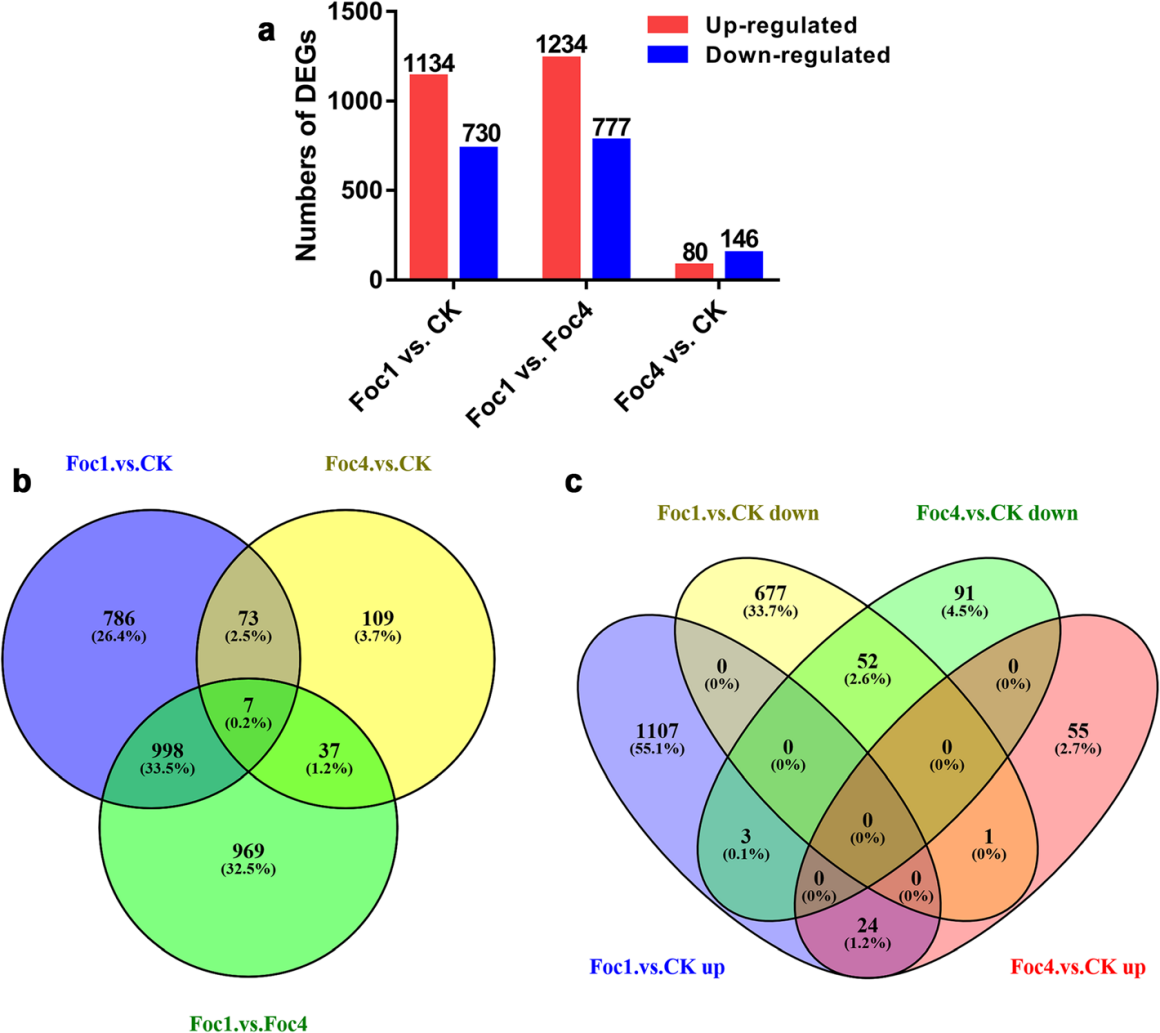
DEGs of ‘Brazilian’ during early infection by Foc. **a** up and down-regulated genes in different compared groups. **b**-**c** Venn diagram analysis of DEGs in three compared groups

To study the gene expression of ‘Brazilian’ roots after infected with Foc, a pairwise comparison was performed between libraries to determine DEGs. The nine libraries were analyzed to determine the number of FPKM of clean reads (Additional file 1: Table S1). Genes with FDR ≤ 0.05 and fold-change ≥1 were considered as differentially expressed compared with the control. A total of 1864 DEGs were identified after Foc1 infection (Additional file 1: Table S2). These DEGs included 1134 up- and 730 down-regulated genes (Fig. 1a), among which 786 genes were specifically expressed (Fig. 1b). In the Foc4 vs. CK group, 226 genes were significantly differentially expressed (Additional file 1: Table S2), including 80 up- and 146 down-regulated genes (Fig. 1a), among which 109 genes were specifically expressed (Fig. 1b). DEGs were dramatically changed in the Foc1 vs. Foc4 group, wherein 2011 DEGs were identified, including 1234 up- and 777 down-regulated genes (Fig. 1a), among which 969 were specifically expressed (Fig. 1b). Further analysis revealed that 80 DEGs were commonly shared in Foc1- and Foc4-responsive genes, of which 24 DEGs were up-regulated, 52 DEGs were down-regulated, and four DEGs showed the opposite expression trends (Fig. 1c).

**Fig. 2:**
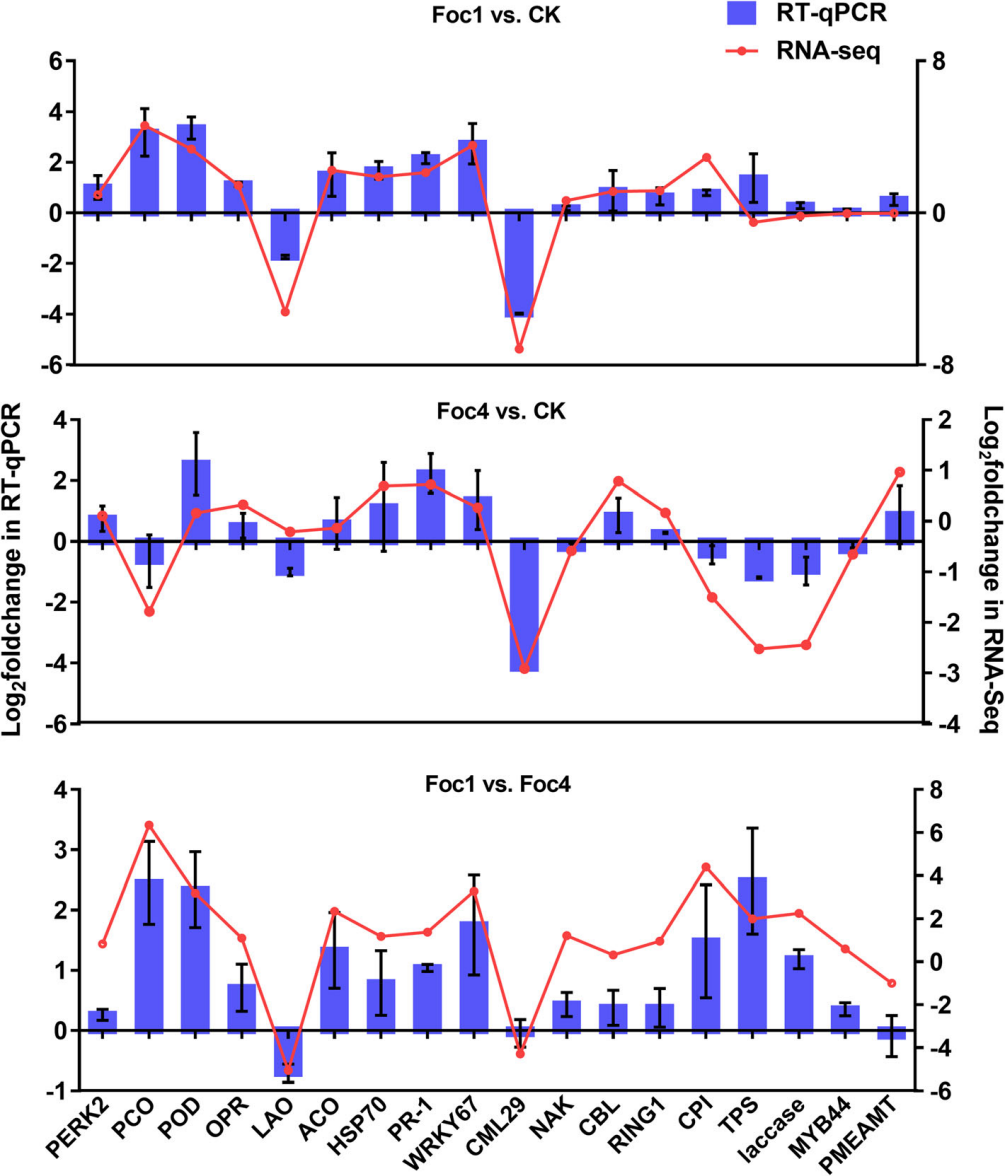
Comparison of the different expression trends among 18 genes in three compared groups using both RT-qPCR and RNA-Seq. Data represent an average of three biological replicates. Error bars are standard deviation. The details description of the 18 genes are shown in Table S1

To verify the RNA-Seq results, 18 DEGs involved in redox homeostasis, phytohormones, signal transduction, and secondary metabolism (the details as shown in Additional file 1: Table S3) were randomly selected for RT-qPCR analysis. The results showed that the expression trends of the selected 18 genes except for three genes (*TPS* in Foc1 vs. CK group, *LAO* in Foc4 vs. CK group and *CML29* in Foc1 vs. Foc4 group) were consistent with the RNA-seq analysis (Fig. 2). This result suggested that the RNA-seq data were reliable.

### GO and KEGG function annotation of DEGs

GO functional analysis showed that DEGs in the Foc1 vs. CK group were enriched into 712 GO terms, of which six were significantly enriched, and DEGs in the Foc4 vs. CK group were enriched into 273 GO terms, of which 16 were significantly enriched, whereas DEGs in the Foc1 vs. Foc4 group were enriched into 729 GO terms without significantly enriched terms (Table 2, Additional file 1: TableS2).

**Table 2:** Statistics of GO terms with significant enrichment of DEGs in three compare groups

Category	GO ID	Description	GeneRatio	pva	padj
Foc1 vs. CK					
MF	GO:0020037	heme binding	42/925	0.00	0.03
MF	GO:0046906	tetrapyrrole binding	42/925	0.00	0.03
MF	GO:0019842	vitamin binding	15/925	0.00	0.03
MF	GO:0005506	iron ion binding	34/925	0.00	0.04
MF	GO:0030170	pyridoxal phosphate binding	13/925	0.00	0.04
MF	GO:0070279	vitamin B6 binding	13/925	0.00	0.04
Foc4 vs. CK					
BP	GO:0006412	translation	14/84	0.00	0.00
BP	GO:0043043	peptide biosynthetic process	14/84	0.00	0.00
BP	GO:0043604	amide biosynthetic process	14/84	0.00	0.00
BP	GO:0006518	peptide metabolic process	14/84	0.00	0.00
BP	GO:0043603	cellular amide metabolic process	14/84	0.00	0.00
BP	GO:0006457	protein folding	5/84	0.00	0.02
CC	GO:0005840	ribosome	14/36	0.00	0.00
CC	GO:0030529	intracellular ribonucleoprotein complex	14/36	0.00	0.00
CC	GO:1990904	ribonucleoprotein complex	14/36	0.00	0.00
CC	GO:0043228	non-membrane-bounded organelle	14/36	0.00	0.00
CC	GO:0043232	intracellular non-membrane-bounded organelle	14/36	0.00	0.00
MF	GO:0003735	structural constituent of ribosome	14/100	0.00	0.00
MF	GO:0005198	structural molecule activity	14/100	0.00	0.00
MF	GO:0020037	heme binding	11/100	0.00	0.00
MF	GO:0046906	tetrapyrrole binding	11/100	0.00	0.00
MF	GO:0051082	unfolded protein binding	4/100	0.00	0.01

*GeneRatio* is the ratio of the number of genes enriched to the GO category and the number of differential genes, *MF* Molecular Function, *BP* Biological Process, *CC* Cellular Component

KEGG enrichment analysis revealed that the DEGs in the three comparison groups were enriched in 102, 45, and 106 pathways, respectively, of which 10, 1 and 6 pathways were significantly enriched (Additional file 1: Table S2). A total of 13 KEGG pathways in the three comparison groups showed significant enrichment, of which four pathways were common significantly enriched in the Foc1 vs. CK and Foc1 vs. Foc4 groups, as follows: phenylalanine metabolism; α-linolenic acid metabolism; phenylalanine; and biosynthesis of tyrosine and tryptophan, ubiquinone, and other terpenoid quinones (Table 3). Intriguingly, the pathway of plant hormone signal transduction was significantly enriched only in the Foc1 vs. Foc4 group, and the number of enriched genes (45) was the highest among all enrichment pathways. The results showed that the number of DEGs enriched in the disease-resistant pathways after Foc1 infection was significantly higher than that after Foc4 infection.

**Table 3:** Distribution of DEGs in significantly enriched KEGG pathways in three compare groups

Map ID	Pathway name	Foc1 vs. CK		Foc4 vs. CK		Foc1 vs. Foc4	
		The number of DEGs	Padj	The number of DEGs	Padj	The number of DEGs	Padj
Mus00400	Phenylalanine, tyrosine and tryptophan biosynthesis	15	0.00			12	0.02
Mus04141	Protein processing in endoplasmic reticulum	34	0.02	/		14	0.00
Mus00360	Phenylalanine metabolism	12	0.02	/		/	/
Mus00960	Tropane, piperidine and pyridine alkaloid biosynthesis	7	0.02	/		/	/
Mus00592	alpha-Linolenic acid metabolism	10	0.02	/		12	0.00
Mus01230	Biosynthesis of amino acids	36	0.02	/			
Mus00130	Ubiquinone and other terpenoid-quinone biosynthesis	10	0.03	/		10	0.03
Mus00100	Steroid biosynthesis	9	0.03	/		/	/
Mus00480	Glutathione metabolism	16	0.03	/		/	/
Mus00270	Cysteine and methionine metabolism	18	0.03	/		/	/
Mus03010	Ribosome	/	/	18	0.00	/	/
Mus04075	Plant hormone signal transduction	/	/	/	/	45	0.02
Mus01212	Fatty acid metabolism	/	/	/	/	14	0.02

### Biotic stress overview pathway analysis

**Fig. 3:**
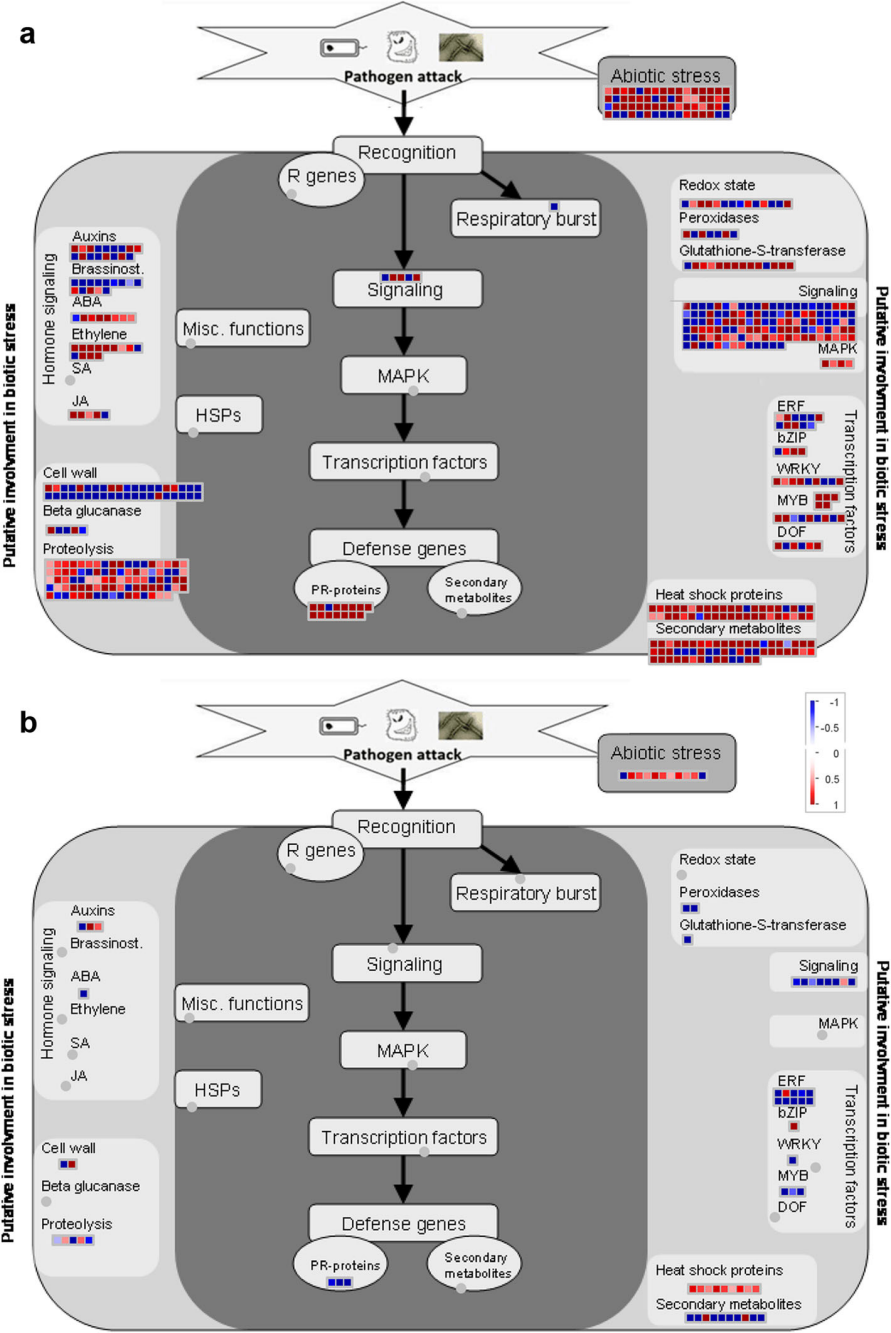
Biotic stress pathway analyses of DEGs in ‘Brazilian’ during early infection by Foc. Biotic stress overview finished with installed toolkit in the MapMan after integration of log_2_ (fold change) data of all detected DEGs in ‘Brazilian’ root after infection with both of Focs. Red boxes mean up-regulated genes and green mean down-regulated genes. **a** 48 h after Foc1 infection; **b** 48 h after Foc4 infection

To further understand difference in the defense response of ‘Brazilian’ plants after infection with Foc1 and Foc4, MapMan program (version 3.5.1) was employed to analyze the change in the transcription of biological stress-related DEGs. As shown in Fig. 3, compared with mock-inoculated control, the types and numbers of DEGs induced by Foc1 infection were significantly more than those induced by Foc4 infection. Intriguingly, except for one down-regulated pathogenesis related protein (PR) gene, the remaining PR protein genes were up-regulated in Foc1 vs. CK group, while three PR protein genes were down-regulated in Foc4 vs. CK group. In addition, some TFgenes, such as MYB, were all up-regulated in the Foc1 vs. CK group, and the expression patterns of other TFs and several secondary metabolism-related genes in Foc1 vs. CK and Foc4 vs. CK groups were also significantly different (Fig. 3).

Finally, we observed that a group of genes associated with the hormones brassinolide, ethylene, and jasmonic acid; four genes associated with MAPK; and 14 genes associated with redox state were differentially expressed only after infection with Foc1 (Fig. 3a). The coregulated genes after infection with Foc1 and Foc4 were related to auxin signal transduction, pathogen-related proteins, proteolysis, and redox homeostasis (Fig. 3). Further analysis found that these genes that were differentially expressed in the Foc1 vs. CK and Foc4 vs. CK groups also showed differential expression in Foc1 vs. Foc4 group (Additional file 2 Fig. S2).

### Metabolism overview and flavonoid pathway analysis

**Fig. 4:**
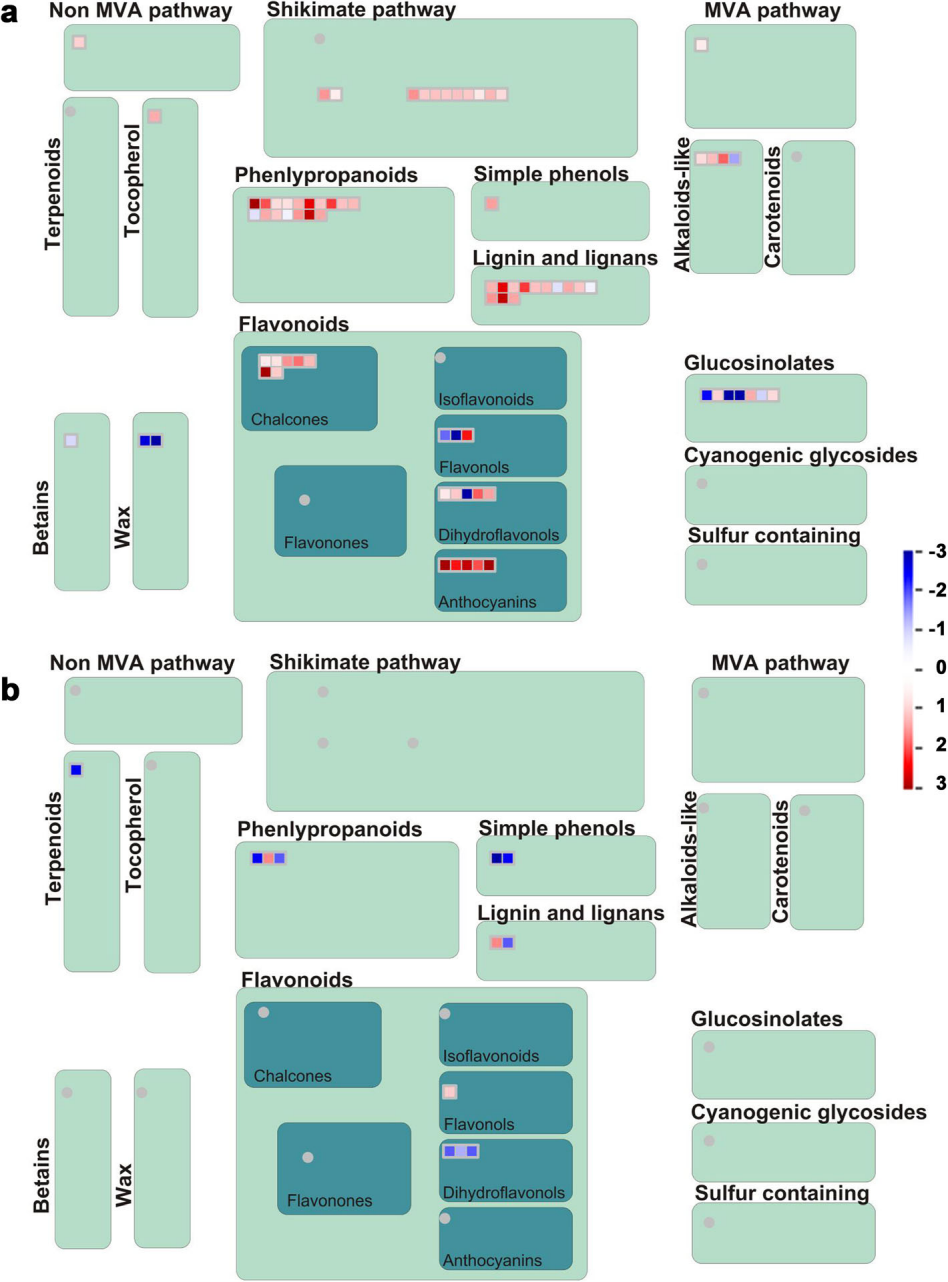
Secondary metabolism pathway analyses of DEGs in ‘Brazilian’ during early infection by Foc. Secondary metabolism pathway analysis of the DEGs was performed using MapMan software. Red boxes mean up-regulated genes and green mean down-regulated genes. **a** 48 h after Foc1 infection; **b** 48 h after Foc4 infection

The above analysis showed that metabolism-related DEGs changed significantly after Foc infection. Thus, a summary of metabolism and secondary metabolic pathways analysis was carried out by MapMan software (Fig. 4, Additional file 2: Fig. S3). A total of 261 DEGs after Foc1 infection were concentrated in metabolism overview pathways. These DEGs were mainly concentrated in secondary metabolic pathways (51), lipids (39), and cell wall (39) (Additional file 2: Fig. S3A). By contrast, only 37 DEGs after Foc4 infection were concentrated mainly in secondary metabolic pathways (10) and lipids (6) (Additional file 2: Fig. S3B). While compared with Foc4 infection, a total of 305 DEGs after Foc1 infection were concentrated in metabolism overview pathways (Additional file 2: Fig. S3C). In secondary metabolism pathway, DEGs participated in phenylpropanoids, lignin, flavonoids, and simple phenols biosynthesis was drastically differed after infection with both races (Fig. 4).

The results of the secondary metabolism pathway analysis showed that the flavonoid pathway had notably changed. As shown in Fig. 4, 20 DEGs in the whole flavonoid biosynthesis pathway were activated. Among these DEGs, five anthocyanin-related genes were all up-regulated after Foc1 infection, whereas only four DEGs related to flavonoid biosynthesis were changed, among which three dihydroflavonol genes (*LOC103975883*, *LOC103998816*, and *LOC103972494*) were down-regulated after Foc4 infection (Fig. 4b). In addition, seven genes involved in the biosynthesis of glucosinolates were expressed exclusively after Foc1infection, whereas one gene involved in terpenoid biosynthesis was expressed exclusively after Foc4 infection (Fig. 4). Intriguingly, all these DEGs were also changed in Foc1 vs. Foc4 groups (Additional file 2: Fig. S4). These results indicated that these DEGs may play an important role in protecting plants from biological stress.

### Lignin biosynthesis pathway related DEGs in response to Foc infection

**Fig. 5:**
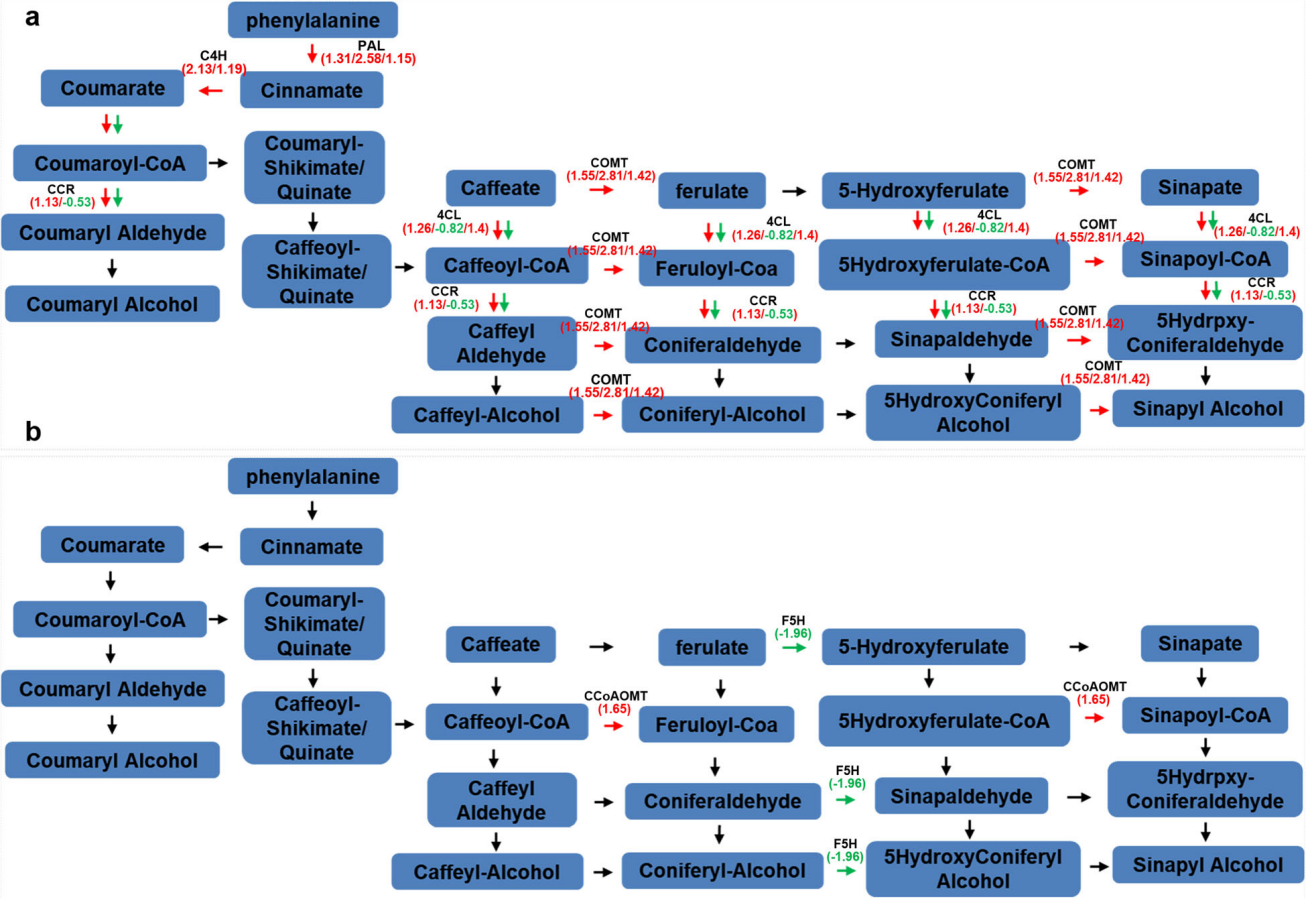
Lignin pathway analyses of DEGs in ‘Brazilian’ during early infection by Foc*.* Analysis of the lignin (phenylpropanoid biosynthesis) pathway of DEGs was performed using MapMan software. Red arrows mean up-regulated genes and green mean down-regulated. Black ones mean no change. **a** 48 h after Foc1 infection; **b** 48 h after Foc4 infection. The pathway frames are from the MapMan software database

The phenylalanine metabolic pathway was a significantly enriched pathway in the KEGG pathway enrichment analysis (Additional file 2: Fig. S5). This pathway also significantly changed in MapMan analysis. As shown in Fig. 5, compared with genes in the mock control, three phenylalnine ammonialyase (*PAL*) genes (*LOC103983874*, *LOC103971627* and *LOC103985827*), two cinnamic acid 4-hydroxylase (*C4H*) genes (*LOC103997903* and *LOC103992160*), two 4-coumaroyl-CoA synthase (*4CL*) genes (*LOC103972208* and *LOC103980720*), one cinnamyl-coenzyme A reductase (*CCR1*) gene (*LOC103984417*), and three caffeic acid 3-O-methyltransferase (*COMT*) genes (*LOC103971889*, *LOC103973932*, and *LOC103971925*) were up-regulated, and *4CL* gene (*LOC103986414*) and one *CCR1* gene (*LOC103982025*) were down-regulated after Foc1 infection, which could lead to coumaryl aldehyde and coniferaldehyde accumulation (Fig. 5a). And all these gene were up-regulated in Foc1 vs. Foc4 group (Additional file 2: Fig. S6). However, only one caffeoyl-CoA O-methyltransferase (*CCoAOMT*) gene (*LOC103973589*) was enhanced expression, and one ferulate 5-hydroxylase *(F5H*) gene (*LOC103972494*) was down-regulated after Foc4 infection (Fig. 5b).

**Fig. 6:**
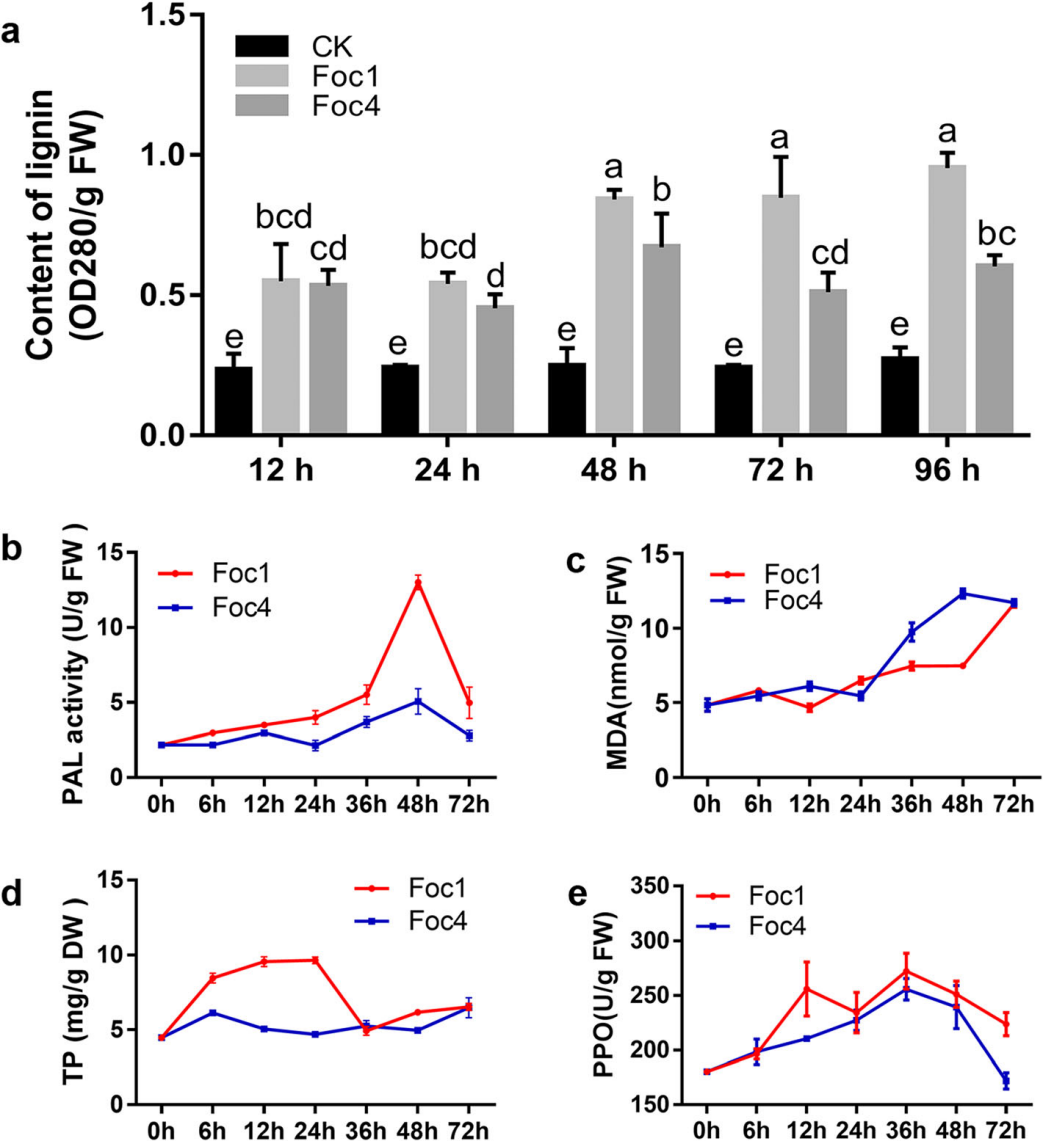
Determination of lignin and phenolic compounds in ‘Brazilian’ roots during early infection by Foc. **a** Lignin content*,* (**b**) PAL activity, (**c**) Malondialdehyde (MDA) content, (**d**) Total phenolics (TP*)* content and (**e**) Polyphenol oxidase (PPO) activity

To confirm the changes in phenylpropanoids after Foc infection, the content of phenylpropanoids (lignin, PAL, and MDA) was determined (Fig. 6). Compared with those after inoculation with the mock control, lignin content and PAL activity increased after inoculation with the two Foc races; however, lignin content after Foc1 infection was significantly higher than that after Foc4 infection (Fig. 6a, b). In addition, Malondialdehyde (MDA) content did not significantly differ at 0-24 hai with Foc1 and Foc4, but was significantly higher at 36–48 hai with Foc4 infection than Foc1 infection. It reached a stable level at 72 hai with Foc1 and Foc4 but remained higher than that after treatment with the mock control (Fig. 6c). The total phenolics (TP) content after Foc1 infection showed a gradual increase and then returned to the mock control level, reaching the highest value at 24 h; after Foc4 infection, the TP content slightly increased at 6 and 72 hai with Foc4 but did not differ at other time points compared with that under inoculation with the mock control. Overall, the TP content in ‘Brazilian’ roots after Foc1 infection was higher than that after Foc4 infection (Fig. 6d). Polyphenol oxidase (PPO) activity first increased and then decreased after Foc1 and Foc4 infection, reaching the highest value at 36 h (Fig. 6e). These results were consistent with the results of our previous analysis.

### Hormone signal pathway related DEGs in response to Foc infection

**Fig. 7:**
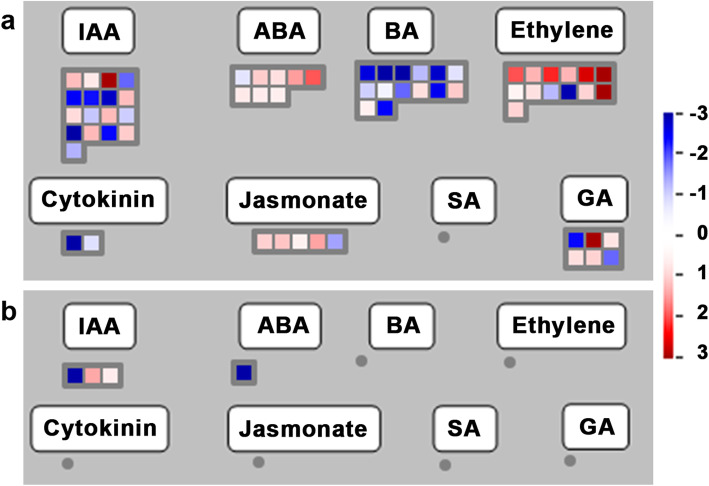
Plant hormone signal pathway analyses of DEGs in ‘Brazilian’ during early infection by Foc*.* Plant hormone signal pathway analyses were performed using MapMan software. Red boxes mean up-regulated genes and green mean down-regulated. **a** Foc1 vs. CK; **b** Foc4 vs. CK

Several hormone-related genes were significantly differentially expressed during the early stages of Foc infection. 17 genes (eight up- and nine down-regulated genes) and three genes (two up- and one down-regulated genes) were associated with the IAA pathway at 48 hai with Foc1 and Foc4, respectively (Fig. 7). While 25 genes (13 up- and 12 down-regulated genes) associated with the IAA pathway were differentially expressed in Foc1 vs. Foc4 group (Additional file 2: Fig. S7). After Foc1 infection, eight genes associated with the ABA pathway (including seven up- and one down-regulated genes) were differentially expressed. However, after Foc4 infection, only one gene associated with ABA was down-regulated. 40 and 52 genes associated with BA, ET, CTK, JA, and GA pathways were differentially expressed in Foc1 vs. CK and Foc1 vs. Foc4 groups, respectively, but were unaffected after Foc4 infection (Fig. 7, Additional file 2: Fig. S7). These results indicated that plant hormone-related signaling pathways may have an indispensable role in early defense response.

We observed that five DEGs related to hypersensitive cell death (*LOC103971926*, *LOC103985684*, *LOC103997880*, *LOC103989280*, and *LOC103971744*) were enhanced expression after Foc1 infection but were unaffected after Foc4 infection compared with those after treatment with the mock control (Additional file 1: Table S4). We speculate that these DEGs may have a critical role in mediating ‘Brazilian’ resistance to Foc.

### Other defense-related pathways analysis

During plant and pathogen interaction, the pathogen can secrete effectors to interfere in host’s defense response. Meanwhile, the host also can produce specific receptors to recognition effectors to inhibit pathogen infection. We observed that 52 RLK genes significantly changed at 48 h after Foc1 infection, whereas only one RLK gene was differentially expressed after Foc4 infection (Additional file 2: Fig. S8).

MapMan analysis showed that many genes encoding E3 ubiquitin-related HECT and FBOX protein were differentially expressed after Foc1 infection, and only one gene encoding HECT protein and one FBOX protein were differentially expressed after Foc4 infection (Additional file 2: Fig. S9). In addition, many heat shock proteins, such as HSP18.1, HSP16.9 and HSP70, were also up-regulated after Foc infection, but the number of the genes up-regulated was much higher after Foc1 infection than that after Foc4 infection (Additional file 1: Table S2).

Large enzyme families, such as cytochrome P450, oxidases, nitrilases, UDP glycosyltransferases, glutathione-S-transferases, GDSL-lipases, and peroxidases, also changed after Foc infection. However, the expression of these enzyme-related genes was significantly different after Foc1 and Foc4 infection. For example, 13 cytochrome P450 enzyme family genes (including nine up- and four down-regulated genes) changed after Foc1 infection, whereas six cytochrome P450 enzyme family genes were down-regulated after Foc4 infection (Additional file 2: Fig. S10). The same was true of the expression of peroxidase and glutathione-S-transferase family genes (Additional file 2: Fig. S10). These results suggested that the genes of large enzyme families may also play a key role in ‘Brazilian’ defense against Foc1 and Foc4.

## Discussion

The unique interaction of Foc with banana provides pivotal molecular information for the breeding of resistant banana varieties. Previous studies reported the gene expression profiles for banana-Foc interactions [23] and comparative transcriptomes of Cavendish resistant and susceptible cultivars [16, 18, 19, 21, 22]. Nevertheless, scarce data exist for assessing the differences in the defenses of Cavendish banana varieties, such as ‘Brazilian’, against Foc1 and Foc4. Li et al. [15] and Dong et al. [4] analyzed the process of Foc1 and Foc4 infection in ‘Brazilian’ plants. We performed quantitative proteome analysis to compare the differences between the defenses of ‘Brazilian’ plants against Foc1 and Foc4 infection [24]. In the current study, we further performed comparative transcriptome analysis with the reference genome of banana to screen genes that specifically contributed to Foc resistance during the early stage of infection to further understand the mechanism underlying the resistance of ‘Brazilian’ to two Foc races. The detailed findings are discussed as follows.

### RLK genes

Pathogens can secrete effectors to regulate plant immunity response, whereas plants also can produce RLK proteins to confront with pathogen infection [25]. Some RLK genes of banana respond to Foc infection [16]. Numerous RLK genes in ‘Brazilian’ were differentially expressed after Foc infection (Additional file 2: Fig. S10). For example, 52 RLK genes changed after Foc1 infection, whereas only one proline extension-like receptor kinase (*PERK*) gene (*LOC103994540*) was down-regulated after Foc4 infection. And compared with Foc4 inoculation, 54 RLK genes changed after Foc1 infection (Additional file 2: Fig. S10). Interestingly, some new receptor kinase genes participated in the different resistances of ‘Brazilian’ plants to two races. These genes included one extension (*LOC10391223*) and two DUF26 kinase genes (*LOC103983462*, *LOC103993931*), which were differentially expressed at 48 hai with Foc1. However, no difference was found after Foc4 infection. Given that these RLK are participated in the sensing and perception of Foc1-derived signals [26], they may be crucial for the difference between the resistance of ‘Brazilian’ against Foc1 and Foc4.

### TFs

Plant TFs are involved in a variety of signaling pathways and play a vital role in plant defense against pathogens. For example, Liu et al. [27] indicated that the expression of *SpMYB* was significantly induced in *Arabidopsis thaliana* after infection with *F. oxysporum*. Jin et al. [28] reported that the *CAPTI1* gene regulates the pepper defense response against *P. capsici*. In banana, Li et al. [18] found that six WRKY family TFs and two ethylene-responsive TFs are up-regulated in the Foc-resistant Cavendish cultivar. Bai et al. [18] suggested that three WRKY TFs showed different expression patterns in resistant and susceptible Cavendish banana varieties after Foc4 infection. Zhang et al. [22] observed that the expression levels of *WRKY4*, *WRKY22*, *WRKY25*, and *WRKY26* were two fold higher in the resistant variety ‘Pahang’ than in ‘Brazilian’ under untreated conditions.

In the present study, we identified 775 and 78 TFs in ‘Brazilian’ plants at 48 hai with Foc1 and Foc4, respectively (Additional file 1: Table S5). These results indicated that the gene transcriptional changes in ‘Brazilian’ induced by Foc1 infection were significantly stronger than those induced by Foc4 infection. Specifically, nine WRKY genes, including *WRKY72*, *WRKY72 X1*, *WRKY43*, *WRKY75*, *WRKY43 X1*, *YPTM2 X1*, *WRKY57*, *WRKY71*, and *WRKY75 X2*, which are involved in plant–pathogen pathways, were differentially expressed in the Foc1 vs. CK group and only one *WRKY43 X1* (*LOC103988831*) was strongly down-regulated in the Foc4 vs. CK group. In addition, 13 WRKY genes were differentially expressed in the Foc1 vs. Foc4 group. These results suggest that the expression of these *WRKY* genes may be related to the difference between the defense mechanisms of ‘Brazilian’ plants against Foc1 and Foc4. Furthermore, two possible *MaWRKY43* and *MaWRKY67* were exclusively up-regulated in ‘Brazilian’ plants after Foc1 infection but were unchanged after Foc4 infection. These results indicate that *MaWRKY43* and *MaWRKY67* are important TFs that determine the basal and induced resistance of ‘Brazilian’ to Foc1. Other potential TF candidates supporting ‘Brazilian’ resistance to Foc1 infection may be downy mildew resistance and ethylene-responsive transcription factor ERF096-like.

NAC TFs are one of the largest families of plant-specific TFs and play diverse roles in plant development and biotic or abiotic stress. Feng et al. [29] suggested that *NAC21/22* was associated with the susceptibility of wheat to diseases. Chen et al. [30] reported that *SmNAC* negatively regulates eggplant resistance to bacterial wilt. Niu et al. [16] suggested that *NAC* domain-containing protein 68-like contributed to Cavendish banana cultivar ‘Yueyoukang No. 1’ resistance to Foc. In current study, five, six NAC TFs were all up-regulated in the Foc1 vs. CK group and Foc1 vs. Foc4 group, respectively, but not differently expressed in the Foc4 vs. CK group (Additional file 1: Table S5). These results indicate that ‘Brazilian’ NAC could play a substantial role in the defense difference against Foc1 and Foc4.

### Classical defense-related genes

Several classical defense-related genes were enhanced expression in ‘Brazilian’ after Foc1 infection but were unaffected after Foc4 infection. For example, cytochrome P450, a member of the JA pathway [31], plays a crucial role in plant defense against pathogens through synthesizing lignin and defense compounds [32]. Yang et al. [33] reported that heterologous expression of *StoCYP77A2* gene enhanced the tobacco resistance to *Verticillium dahliae*. Niu et al. [16] and Zhang et al. [22] suggested that the cytochrome P450 gene exhibits up-regulation in a resistant Cavendish banana cultivar than in a susceptible Cavendish banana cultivar after Foc4 infection. Thus, we hypothesize that the high expression of the cytochrome P450 gene after Foc1 infection may contribute to the resistance of ‘Brazilian’ against Foc1.

PR protein accumulation is one of the main characteristics of plant response to biological or abiotic stress. In present study, five PR-1 like genes (*LOC103977653*, *LOC103975648*, LOC103982935, *LOC103977651*, and *LOC103998084*) and one PR-4 (*LOC103989972*) belonging to chitinases were exclusively up-regulated in ‘Brazilian’ plants after Foc1 infection. Surprisingly, PR genes were unchanged after Foc4 infection. Chitinases are the first line of defense responses in plants, which can hydrolyze the major components of the fungal cell wall and produce chitin oligomers eliciting the plant defense response [34]. They have been shown to have a crucial role in Cavendish banana cultivar defense against Foc infection [18]. Combined with our results, we believe that the up-regulation of PR genes possibly contribute to the resistance difference against Foc1 and Foc4 at the early infection stage in ‘Brazilian’ plants.

### Flavonoid biosynthesis pathway genes

Flavonoids, a group of secondary metabolites, play a significant role in plant defense against pathogens [35]. Siemens et al. [36] and Zhao et al. [37] indicated that numerous genes involved in flavonoid pathway were enhanced expression in *Arabidopsis thaliana* after infection with *Plasmodiophora brassicae*, as a consequence, the content of flavonoid was accumulated in clubroot galls of *Arabidopsis*. Only two CHS genes and a LDOX gene involved in flavonoid biosynthesis were up-regulated in the roots of the resistant banana variety ‘Guijiao 9’ during incompatible banana–Foc4 interaction (Sun et al., 2019). In the current study, 20 DEGs were identified after Foc1 infection, but only four DEGs were activated after Foc4 infection. Enzymes involved in flavonoid biosynthesis were preferentially up-regulated in ‘Brazilian’ plants after Foc1 infection. Fan et al. [5] reported that flavonoid content of ‘Brazilian’ roots at 48 hai with Foc1 was significantly higher than that at 48 hai with Foc4. This result indicated that our RNA-Seq data were accurate. Collectively, the results suggest that the pathway of flavonoid biosynthesis is associated with the response of ‘Brazilian’ plantsto early infection with Foc.

### Genes involved in lignin biosynthesis pathway

Lignification is an effective defense mechanism of plants, which helps plants resist pathogen infection by producing lignin to reinforcement plant cell [38]. Lignin metabolism and production have important roles in response to multiple pathogens, such as the cotton wilt fungus *Verticillium dahliae* [39] and the southern leaf blight and gray leaf spot of maize [40]. Zhang et al. [22] reported that the global expression of most banana enzyme genes, such as *PAL*, *C4H*, *4CL*, *CHS*, *POD*, *HCT*, and *C3*′*H*, was up-regulated in the resistant banana variety ‘Pahang’ at 7 and 14 days after Foc4 infection. The expression of two enzyme-related gene *4CL* and *CAD* in the lignin biosynthesis pathway was induced in the resistant cultivar ‘Guijiao 9’ at 7 days after Foc4infection [21]. In the early stage of infection by Foc1, *PAL*, *C4H, 4CL, CCR1*, and *COMT* were up-regulated, and only one *4CL* and one *CCR1* were down-regulated, whereas *CCoAOMT* was up-regulated, and one *F5H* was down-regulated after Foc4 infection. Meanwhile, we found that the expression of numerous cell wall modification-related genes were also enhanced (Fig. 4 and Additional file 2: Fig. S3). Hammerschmidt et al. [41, 42] reported that lignification could induces systemic resistance in cucumber, and lignin deposition were involved in cucumber defense response against *Cladosporium cucumerinum*. El Modafar and El Boustani [43] suggested that lignin contents are associated with the date palm resistance to *F. oxysporum*. Lignin accumulation regulates the resistance of *Arabidopsis* to *P. brassicae* [37]. Our results showed that lignin began to accumulate at the early infection stage to enhance resistance to Foc (Fig. 6a), suggesting that the lignin biosynthesis may be a crucial component of ‘Brazilian’ defense difference in response to Foc1 and Foc4infection.

### Hormonal changes

Hormone play a key role in plants and pathogens interactions. Foc4 stimulates the synthesis of Cavendish banana plant hormones (mainly JA and ABA), which are essential for banana defense against Foc [18, 19]. In the current study, five JA signal-related genes and eight ABA signal-related genes were differentially expressed in the Foc1 vs. CK group, whereas only one ABA signal-related gene was down-regulated and JA-related genes were unchanged in the Foc4 vs. CK group. Furthermore, similar to previous researchers [18, 19], we did not find significant differences in SA signaling-related genes after infection between two Foc races, indicating that SA does not play a critical role in resistance. Li et al. [18] pointed out that the transcription levels of two ethylene signaling genes in resistant mutant plants are considerably higher than those in wild-type plants. In the current study, thirteen ethylene signaling genes significantly accumulated in ‘Brazilian’ plants after Foc1 infection but were unaffected after Foc4 infection. Interestingly, we also observed that many genes associated with BA, CTK and GA synthesis, metabolism, and transport were differently expressed in ‘Brazilian’ plants after Foc1 infection but not after Foc4 infection. This observation suggest that these genes may also play a significant role in the defense difference of ‘Brazilian’ against Foc1. We speculate that hormones may promote the interaction between banana plants and Foc in the early infection stage. In general, these results suggest that the differential expression of hormonal-related gene is linked to the different patterns in ‘Brazilian’ against infection by both Foc races.

Hypersensitive cell death can help plants successfully resist the infection of biotrophic pathogens, while necrotrophic pathogens have been reported to utilize dead tissues and resist the hypersensitive responses of plant [44]. Zhao et al. [37] revealed that hormone signaling could trigger a hypersensitive response in plants. In the current study, five genes related to plant hypersensitive responses were also changed significantly at 48 h after Foc1 infection but not affected after Foc4 infection (Additional file 1: Table S4). We speculate that the expression of hypersensitive cell death-related genes in ‘Brazilian’ plants at this stage is related to the difference of the resistance of ‘Brazilian’ plants against Foc1 and Foc4. However, further studies are required to verify the speculation.

### Model of ‘Brazilian’-Foc1 and Foc4 interaction

**Fig. 8:**
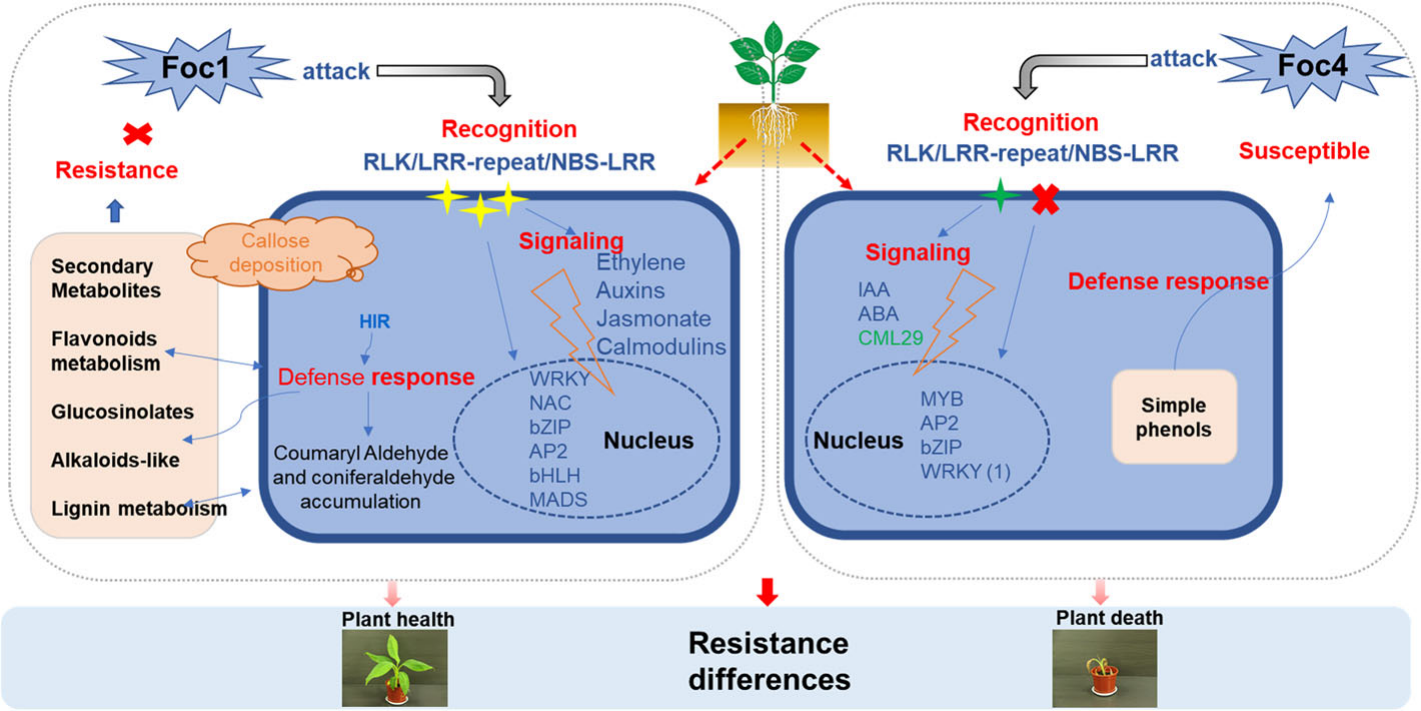
Interaction model of ‘Brazilian’ after infection with Foc1 and Foc4. Graphical representation of Brazilian-Foc interaction at cellular level. Up-regulated resistance proteins are represented with yellow stars, down-regulated resistance proteins are represented with green stars and important steps of the reactions in red. Up-regulated DEGs are in blue and down-regulated DEGs are in green

In combination with the previous analysis above, we outlined a simple model of the interaction between ‘Brazilian’ plants with two Foc races of Foc1 and Foc4 (Fig. 8). In the early infection stage, the response of ‘Brazilian’ plants to Foc4 appears to be weaker and slower than that to Foc1. First, in the recognition stage, the expression of multiple cell membrane receptors and LRR-serine/threonine protein kinases, are up-regulated after Foc1 infection, whereas only one RLK gene *(PERK*, *LOC103994540*) is down-regulated after Foc4 infection. These phenomena indicate that the first layer of immunity (PTI) in plants triggered by Foc4 infection is weaker than that triggered by Foc1 infection. Second, the cellular immune responses induced by the two Foc races after invading ‘Brazilian’ plants are considerably different. For example, after Foc1 infection, a large number of related genes are involved in secondary metabolism, TFs, plant hormones, and signal transduction are differentially expressed. However, after Foc4 infection, only a small part of the TFs and signal transduction-related genes are differentially expressed. Moreover, the amplitude of the up-regulation of these genes after Foc4 infection is weaker than that after Foc1 infection. Third, five genes that may cause hypersensitive cell death are up-regulated after Foc1 infection. Although plant immune response may occur at this infection stage, the related genes are not differentially expressed after Foc4 infection. Therefore, we speculate that a completely different interaction models between ‘Brazilian’ and each of two races of Foc1 and Foc4 exists.

## Conclusions

In the present study, we combined transcriptomics and physiologic approaches to better understand the resistance differences of ‘Brazilian’ defense against Foc1 and Foc4. Our results showed that the changes of gene expression and pathways in ‘Brazilian’ after infection with Foc1 and Foc4 was significantly different. Specifically, the early stages after Foc1 infection can cause differential accumulation and expression of multiple defense-related compounds and defense-related genes, while after Foc4 infection, the changes in defense-related compounds and gene expression were almost unaffected or weakly affected. In addition, the DEGs participating in phenylpropanoid metabolism and cell wall modification were also differentially expressed. We think that these differences contribute to the resistance differences of ‘Brazilian’ to the two Foc races. The results of this study provide new clues for the resistance mechanism of ‘Brazilian’ to Foc1 and Foc4, and provide a new theoretical basis for accelerating the molecular breeding process of bananas.

## Methods

### Plant material and pathogen inoculation

The Cavendish banana cultivar ‘Brazilian’ (*Musa acuminate* L. AAA group) (purchased from the Institute of Fruit Tree Research, Guangdong Academy of Agricultural Sciences) which is susceptible to Foc4 and resistant to Foc1, was selected [7]. The fifth fully developed leaves of banana plants were used for inoculation, a total of ten plants were prepared for each treatment, and each experiment was repeated thrice as biological replicates. The inoculated plants were cultured in a constant temperature light incubator (28 °C, 16 h light and 8 h dark) with a light intensity of 5000 lx. Wild-type Foc1 FJZ3 (VCG01221), Foc4 XJZ2 (VCG01216) and GFP-tagged Foc1 and Foc4 strains were used for the inoculation experiments, and the inoculation protocol referred to a previous method [4].

### RNA extraction

Ten individual ‘Brazilian’ root samples were pooled to one treatment, and each sample was collected in three biological replicates. The total RNA of each root sample was extracted using Trizol reagent (Invitrogen, USA) according to the manufacturer’s protocol. The RNA concentrations were tested by Nanodrop (Bio-Rad, USA), and RNA integrity number (RIN) and 28S/18S were detected using an Agilent 2100 Bioanalyzer (Agilent, USA). After RNA quality evaluation, RNA samples were stored at − 80 °C for later use.

### Library preparation and Illumina sequencing

RNA at 3 μg per sample was prepared to construct cDNA libraries. cDNA libraries were constructed using NEBNext® Ultra™ RNA Library Prep Kit for Illumina® (New England BioLabs, USA) according to the manufacturer’s instructions. Total RNA with RIN > 8 was used for Illumina sequencing. cDNA library preparation and Illumina sequencing were conducted at the Beijing Novogene Company (Beijing, China) according to the standard Illumina instructions. The cDNA libraries were sequenced on an Illumina HiSeq 2000 system with the 150 bp paired-end mode, and the library quality was assessed on the Agilent Bioanalyzer 2100 system.

### Genome mapping and analysis of DEGs

Reads containing adapters and poly-N and low-quality reads were removed from the original raw data (raw reads) to generate clean reads. The clean reads were then compared with the banana reference genome (*M. acuminate* subsp. *malaccensis*) sequences using HISAT aligner software [45]. Then, the sequence results were assessed in accordance with read quality, saturation, alignment, and distribution on banana reference genome [46]. For novel transcript prediction, the mapped reads of each sample were assembled by StringTie (v1.3.3b) (Pertea et al., 2015) in a reference-based approach. Feature Counts v1.5.0-p3 was employed to count the numbers of reads mapped to each gene [47]. The raw read counts for each banana gene were derived and then normalized to fragments per kilobase of transcript per million reads (FPKM) (Mortazavi et al., 2008) and this was used to estimate the expression levels of each gene. Differential expression analysis of two groups (Foc1 vs. CK, Foc4 vs. CK and Foc1 vs. Foc4) was carried out using the DESeq2 R package (1.16.1) based on raw counts of banana genes [48]. The *P*-values were adjusted using the Benjamini and Hochberg’s approach to control false discovery rate (FDR) [49]. Genes with an adjusted *P*-value < 0.05 (padj< 0.05) and the absolute value of Fold change ≥1 obtained from DESeq2 were designated as DEGs.

### Quantitative PCR analysis

RT-qPCR analysis was performed to confirm the gene expression levels shown by Illumina sequencing results, and 18 genes were selected. RT-qPCR was carried out on a CFX96™ Real-Time PCR Detection System (Bio-Rad) using SYBR Premix Ex Taq Kit (TakaRa, JA) following the manufacturer’s protocol. The banana ribosome protein S2 (*RPS2*) gene was used as an internal reference gene [50]. Each experiment was performed in triplicate. Finally, relative gene expression level was quantified by 2^-ΔΔCt^ method [51]. The primers used for RT-qPCR analysis was listed in Additional file 1: Table S3.

### Functional analysis of DEGs

The GO enrichment analysis of the DEGs selected above was implemented by the cluster Profiler R package, in which gene length bias was corrected. GO terms with padj< 0.05 were assigned as significantly enriched by DEGs. Cluster Profiler R package was used to test the statistical enrichment of DEGs in KEGG pathways. In addition, in order to more fully understand the changes in gene expression and pathway, DEGs were further analyzed and visualized using MapMan software [52].

### Phenolic substance determination

To determine whether Foc could induce lignin synthesis in ‘Brazilian’ during infection, the changes in the contents of lignin, TP, and MDA and the activities of PAL and PPO were determined separately with lignin, TP, MDA, PAL, and PPO assay kits (Comin Biotechnology Co., Ltd., Suzhou, China) according to the manufacturer’s protocol. Each experiment was repeated thrice.

## Supplementary Information

**Additional file 1: Table S1.** Gene-FPKM identified in the current study. **Table S2.** Expression profiles and function annotation of the significantly DEGs identified in the study. **Table S3.** Primers used for RT-qPCR analysis. **Table S4.** Expression profiles of the DEGs related to hypersensitive cell death. **Table S5.** TFs identified in the study.

**Additional file 2: Fig. S1.** Expansion and colonization in the roots of ‘Brazilian’ infected respectively with either GFP-tagged isolates of Foc1 or Foc4 at 48 h after inoculation. **Fig. S2.** Biotic stress pathway analyses of DEGs in Foc1 vs. Foc4 group in ‘Brazilian’ at 48 h after infection by Foc. Biotic stress overview finished with installed toolkit in the MapMan after integration of log_2_ (fold change) data of all detected DEGs in ‘Brazilian’ root after infection with both of Focs. Red boxes mean up-regulated genes and green mean down-regulated genes. **Fig. S3.** Metabolism pathway analyses of DEGs in ‘Brazilian’ during early infection with both of Foc races*.* Metabolism pathway analysis of the DEGs was performed using MapMan software. Red boxes mean up-regulated genes and green mean down-regulated genes. (a) Foc1 vs. CK; (b) Foc4 vs. CK; (c) Foc1 vs. Foc4. **Fig. S4.** Secondary metabolism pathway analyses of DEGs in Foc1 vs. Foc4 group in ‘Brazilian’ at 48 h after infection by Foc. Secondary metabolism pathway analysis of the DEGs was performed using MapMan software. Red boxes mean up-regulated genes and green mean down-regulated genes. **Fig. S5.** Phenylalanine metabolism pathway analyses of DEGs in ‘Brazilian’ during early infection with both of Foc races*.* Analysis of the phenylalanine metabolism pathway of DEGs was performed. Red mean up-regulated genes and green mean down-regulated genes. (a) Foc1 vs. CK; (b) Foc1 vs. Foc4. **Fig. S6.** Lignin pathway analyses of DEGs in Foc1 vs. Foc4 group in ‘Brazilian’ after infection by Foc*.* Analysis of the lignin (phenylpropanoid biosynthesis) pathway of DEGs was performed using MapMan software. Red arrows mean up-regulated genes and green mean down-regulated. Black ones mean no change. The pathway frames are from the MapMan software database. **Fig. S7.** Plant hormone signal pathway analyses of DEGs in Foc1 vs. Foc4 group in ‘Brazilian’ after infection by Foc*.* Plant hormone signal pathway analyses were performed using MapMan software. Red boxes mean up-regulated genes and green mean down-regulated. **Fig. S8.** Receptor-like kinases gene analyses of DEGs in ‘Brazilian’ during early infection with both of Foc races*.* Analysis of the receptor-like kinases pathways of DEGs were performed using MapMan software. Red boxes mean up-regulated genes and green mean down-regulated genes. (a) Foc1 vs. CK; (b) Foc4 vs. CK; (c) Foc1 vs. Foc4. **Fig. S9.** Ubiquitin pathway analyses of DEGs in ‘Brazilian’ during early infection with both of Foc races. Analysis of the ubiquitin pathway of DEGs was performed using MapMan software. Red boxes mean up-regulated genes and green mean down-regulated genes. (a) Foc1 vs. CK; (b) Foc4 vs. CK. **Fig. S10.** Large enzyme families analysis of DEGs in ‘Brazilian’ during early infection with both of Foc races. Analysis of the ubiquitin pathway of DEGs was performed using MapMan software. Red boxes mean up-regulated genes and green mean down-regulated genes. (a) Foc1 vs. CK; (b) Foc4 vs. CK.

## Data Availability

The data used and analyzed during the present study are included in the figures and tables of the manuscript. And raw RNA-seq data from nine samples have been deposited into the NCBI Sequence Read Archive (SRA) database under accession SRP229393.

## Abbreviations

DEGs: Differentially expressed genes
FDR: Flase discovery rate
Foc: *Fusarium oxysporum *f. sp. *cubense*
Foc1: *Fusarium oxysporum* f. sp. *cubense* race 1
Foc4: *Fusarium oxysporum* f. sp. *cubense* race 4
FPKM: Fragments per kilobase of transcript per million reads
hai: Hour after inoculation
TFs: Transcription factors
TP: Total phenolics
MDA: Malondialdehyde
PAL: Phenylalnine ammonialyase
PPO: Polyphenol oxidase
RLKs: Receptor-like kinases
