# Supplementary material for: Comparative transcriptome analysis revealed resistance differences of Cavendish bananas to Fusarium oxysporum f.sp. cubense race1 and race4
Source: BMC Genet. 2020 Nov 11;21:122. doi: 10.1186/s12863-020-00926-3 (PMC7657330; doi:10.1186/s12863-020-00926-3)
Supplement: Supplementary file 2 — Additional file 2: Fig. S1. Expansion and colonization in the roots of ‘Brazilian’ infected respectively with either GFP-tagged isolates of Foc1 or Foc4 at 48 h after inoculation. Fig. S2. Biotic stress pathway analyses of DEGs in Foc1 vs. Foc4 group in ‘Brazilian’ at 48 h after infection by Foc. Biotic stress overview finished with installed toolkit in the MapMan after integration of log2 (fold change) data of all detected DEGs in ‘Brazilian’ root after infection with both of Focs. Red boxes mean up-regulated genes and green mean down-regulated genes. Fig. S3. Metabolism pathway analyses of DEGs in ‘Brazilian’ during early infection with both of Foc races. Metabolism pathway analysis of the DEGs was performed using MapMan software. Red boxes mean up-regulated genes and green mean down-regulated genes. (a) Foc1 vs. CK; (b) Foc4 vs. CK; (c) Foc1 vs. Foc4. Fig. S4. Secondary metabolism pathway analyses of DEGs in Foc1 vs. Foc4 group in ‘Brazilian’ at 48 h after infection by Foc. Secondary metabolism pathway analysis of the DEGs was performed using MapMan software. Red boxes mean up-regulated genes and green mean down-regulated genes. Fig. S5. Phenylalanine metabolism pathway analyses of DEGs in ‘Brazilian’ during early infection with both of Foc races. Analysis of the phenylalanine metabolism pathway of DEGs was performed. Red mean up-regulated genes and green mean down-regulated genes. (a) Foc1 vs. CK; (b) Foc1 vs. Foc4. Fig. S6. Lignin pathway analyses of DEGs in Foc1 vs. Foc4 group in ‘Brazilian’ after infection by Foc. Analysis of the lignin (phenylpropanoid biosynthesis) pathway of DEGs was performed using MapMan software. Red arrows mean up-regulated genes and green mean down-regulated. Black ones mean no change. The pathway frames are from the MapMan software database. Fig. S7. Plant hormone signal pathway analyses of DEGs in Foc1 vs. Foc4 group in ‘Brazilian’ after infection by Foc. Plant hormone signal pathway analyses were performed using MapMan software. Re [file 12863_2020_926_MOESM2_ESM.docx]

Supplementary Material


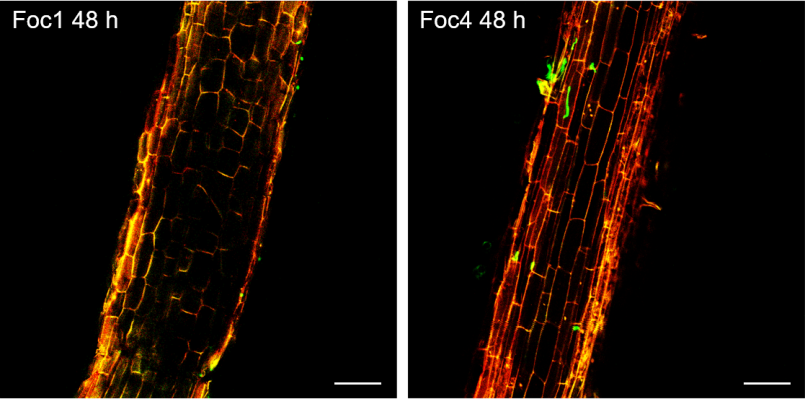


**Additional file 2: FigureS1.**Expansion and colonization in the roots of ‘Brazilian’ infected respectively with either GFP-tagged isolates Foc1 or Foc4 at 48 h after inoculation.


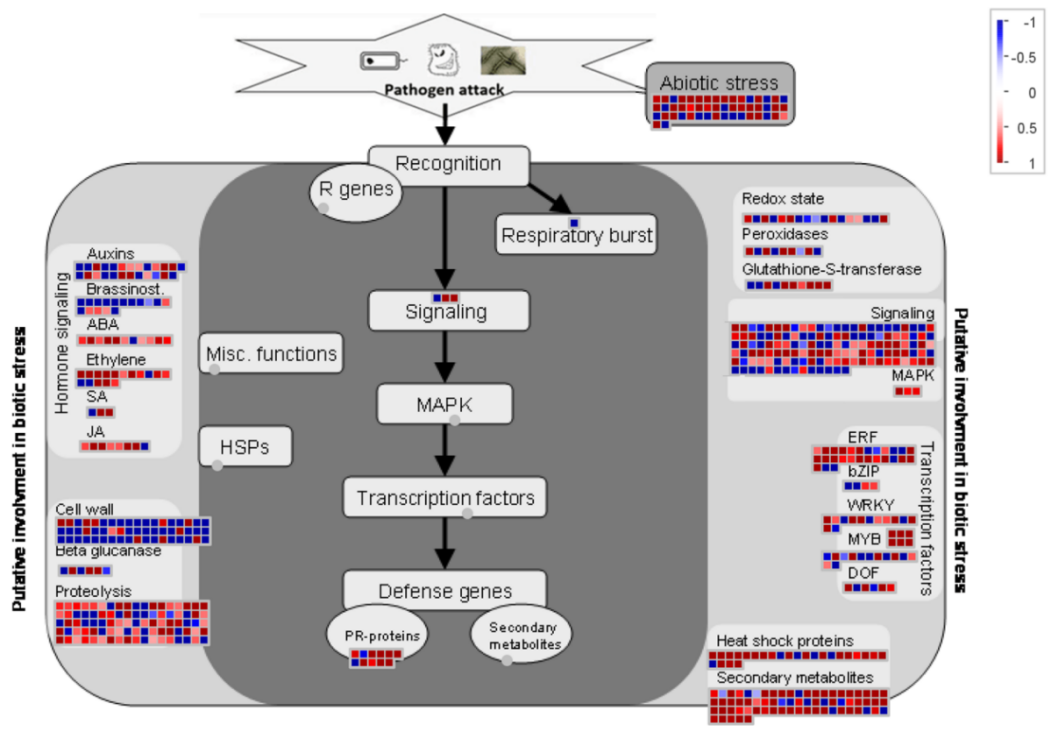


**Additional file 2:FigureS2.**Biotic stress pathway analyses of DEGs in Foc1 vs. Foc4 group in ‘Brazilian’ at 48 h after infection by Foc. Biotic stress overview finished with installed toolkit in the MapMan after integration of log_2_(fold change) data of all detected DEGs in ‘Brazilian’ root after infection with both of Focs. Red boxes mean up-regulated genes and green mean down-regulated genes.


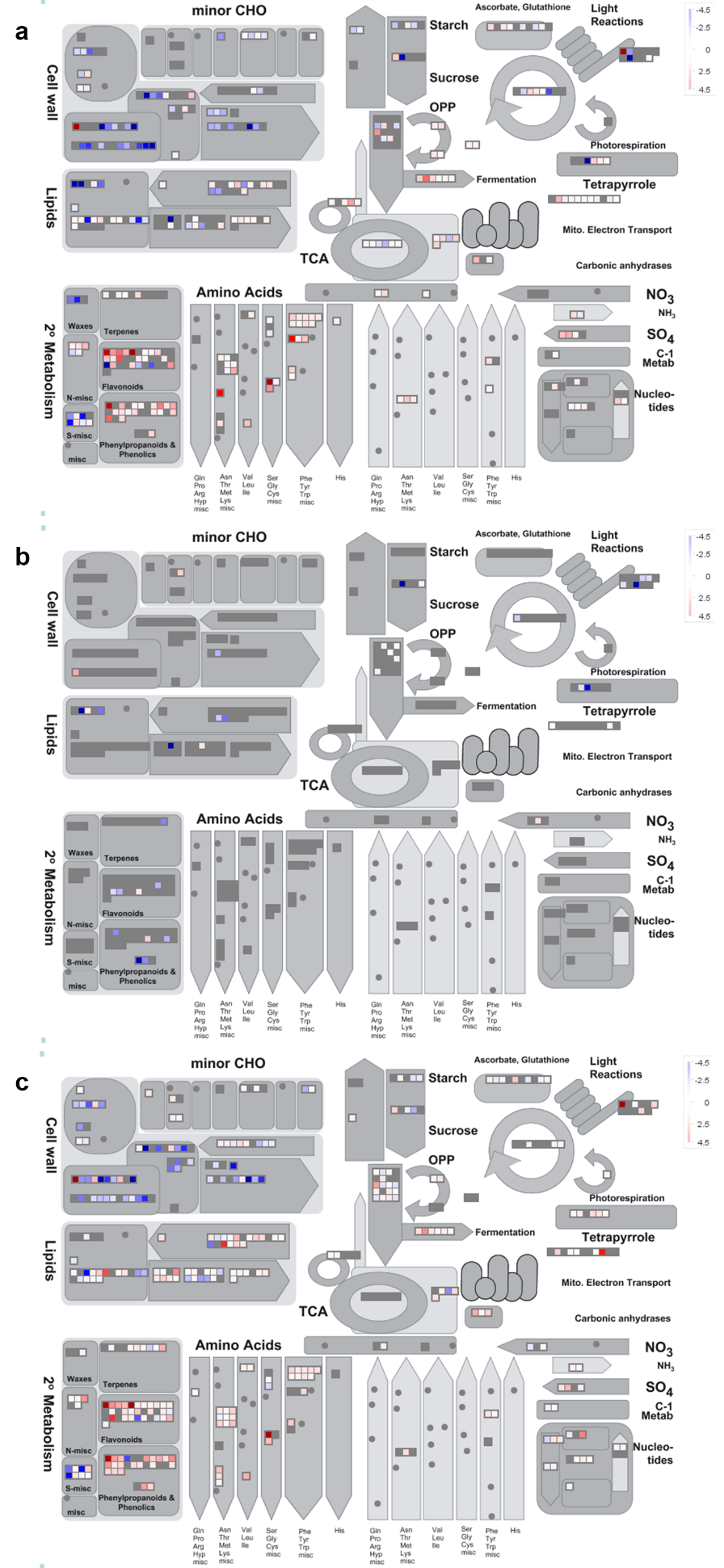


**Additional file 2: Figure S3.** Metabolism pathway analyses of DEGs in ‘Brazilian’during early infection with both of Foc races*.* Metabolism pathway analysis of the DEGs was performed using MapMan software. Red boxes mean up-regulated genes and green mean down-regulated genes. (a) Foc1 vs. CK; (b) Foc4 vs. CK;(b) Foc1 vs. Foc4.


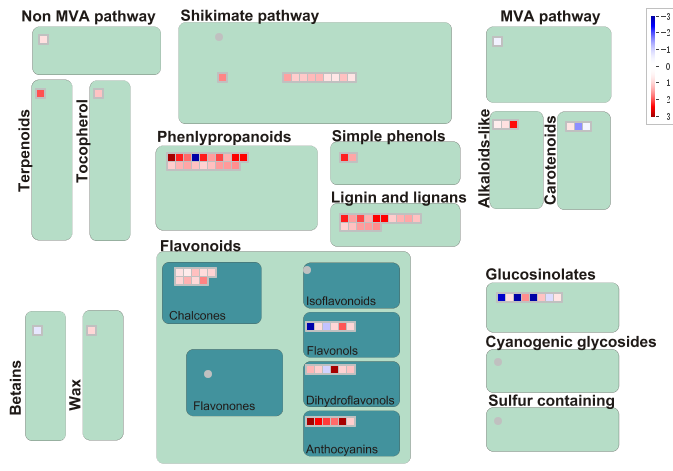


**Additional file 2:FigureS4.**Secondary metabolism pathway analyses of DEGs inFoc1 vs. Foc4 group in ‘Brazilian’ at 48 h after infection by Foc.Secondary metabolism pathway analysis of the DEGs was performed using MapMan software. Red boxes mean up-regulated genes and green mean down-regulated genes.


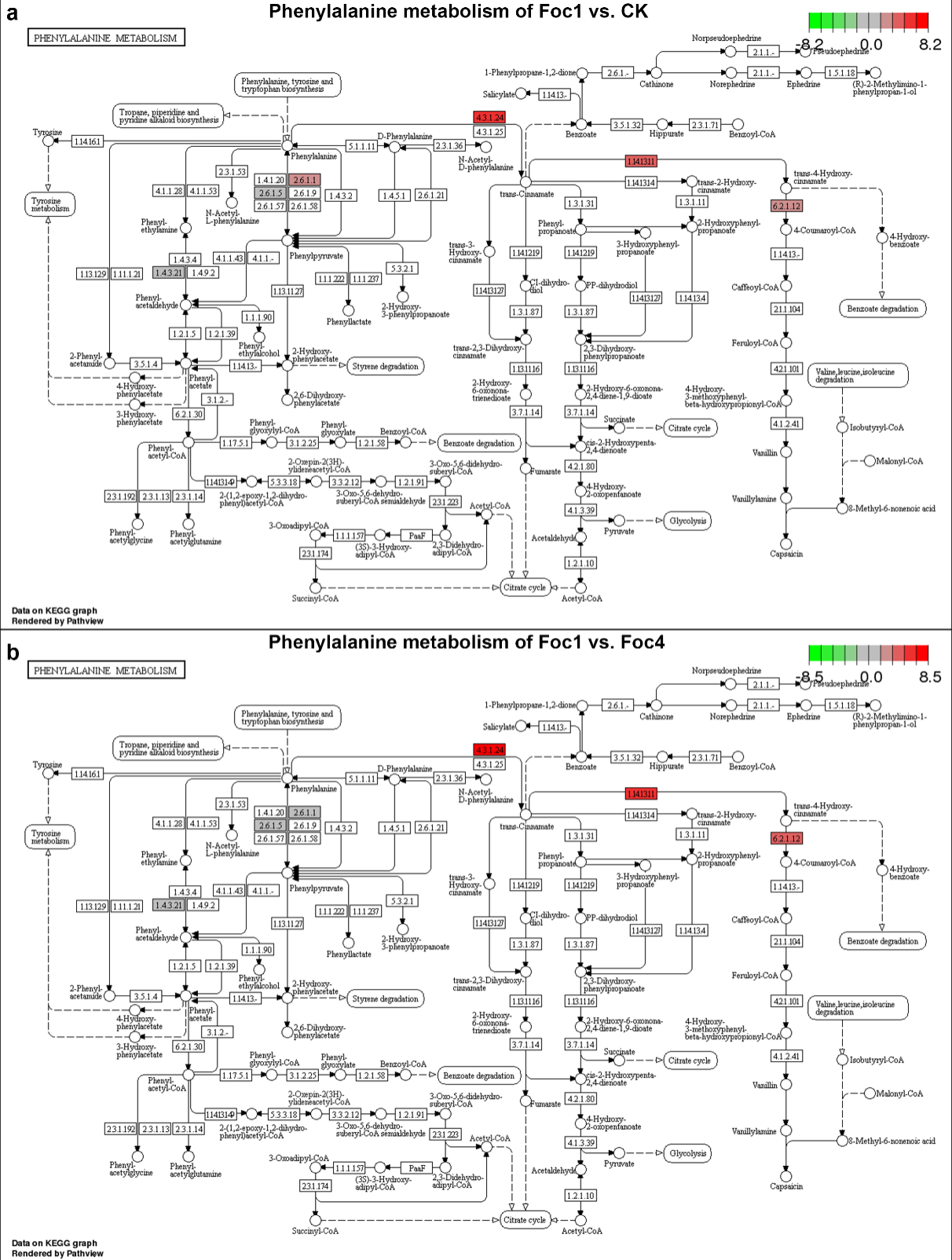


**Additional file 2: Figure S5.** Phenylalanine metabolism pathway analyses of DEGs in ‘Brazilian’ during early infection with both of Foc races*.* Analysis of the phenylalanine metabolism pathway of DEGs was performed. Red mean up-regulated genes and green mean down-regulated genes. (a) Foc1 vs. CK;(b) Foc1 vs. Foc4.


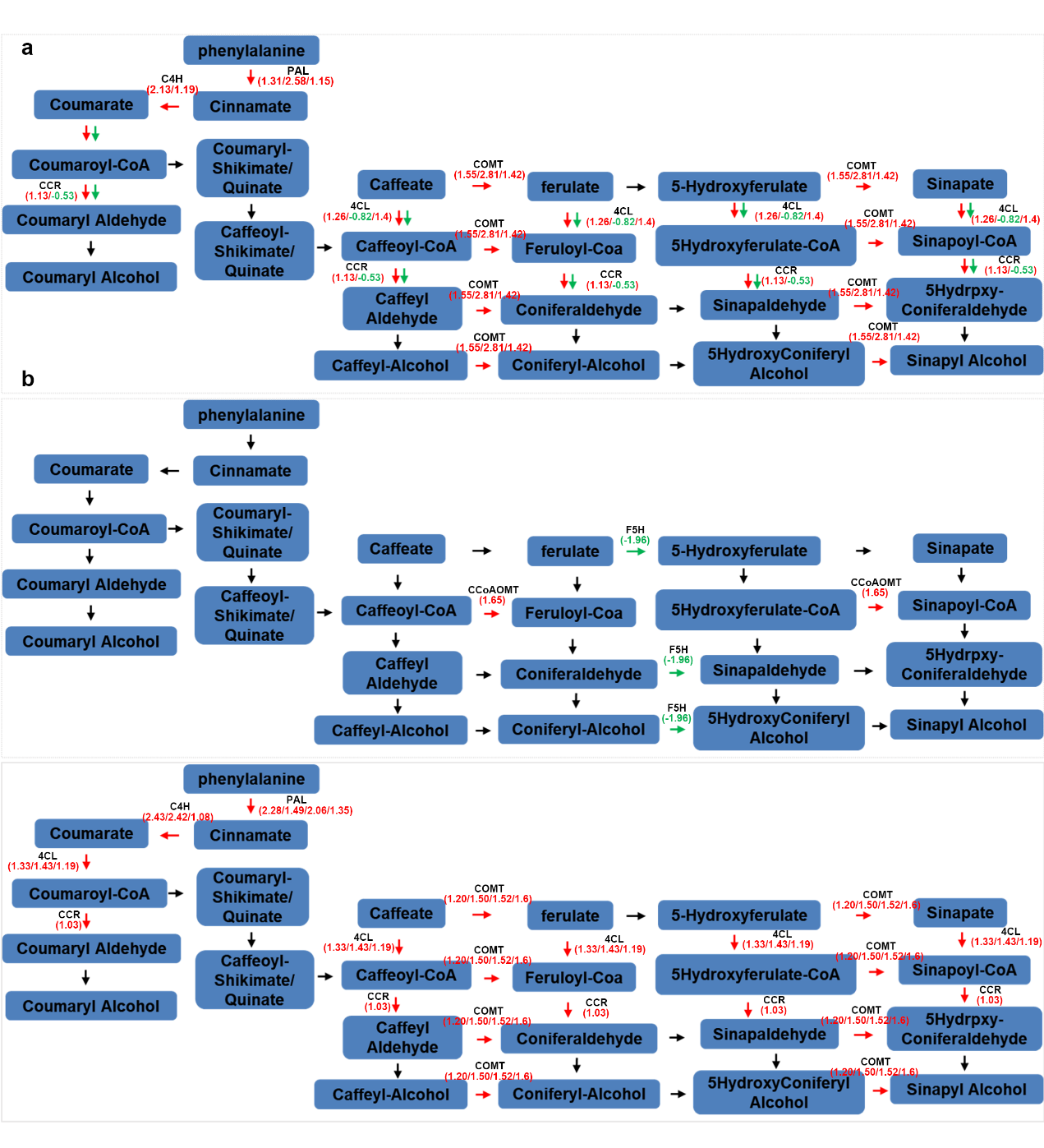


**Additional file 2:FigureS6.**Lignin pathway analyses of DEGs inFoc1 vs. Foc4 group in ‘Brazilian’after infection by Foc*.* Analysis of the lignin (phenylpropanoid biosynthesis) pathway of DEGs was performed using MapMan software. Red arrows mean up-regulated genes and green mean down-regulated. Black ones mean no change. The pathway frames are from the MapMan software database.

**
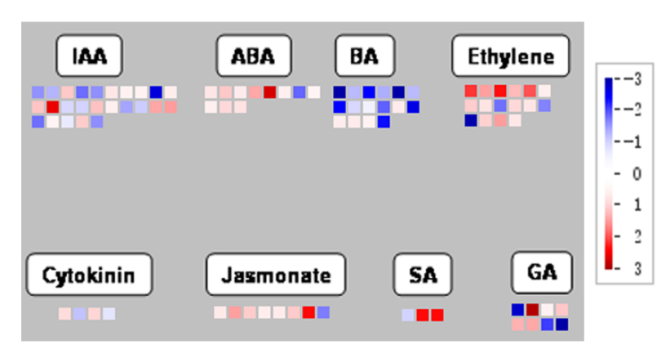
**

**Additional file 2: FigureS7.** Plant hormone signal pathway analyses of DEGs inFoc1 vs. Foc4 group in ‘Brazilian’after infection by Foc*.* Plant hormone signal pathway analyses were performed using MapMan software. Red boxes mean up-regulated genes and green mean down-regulated.


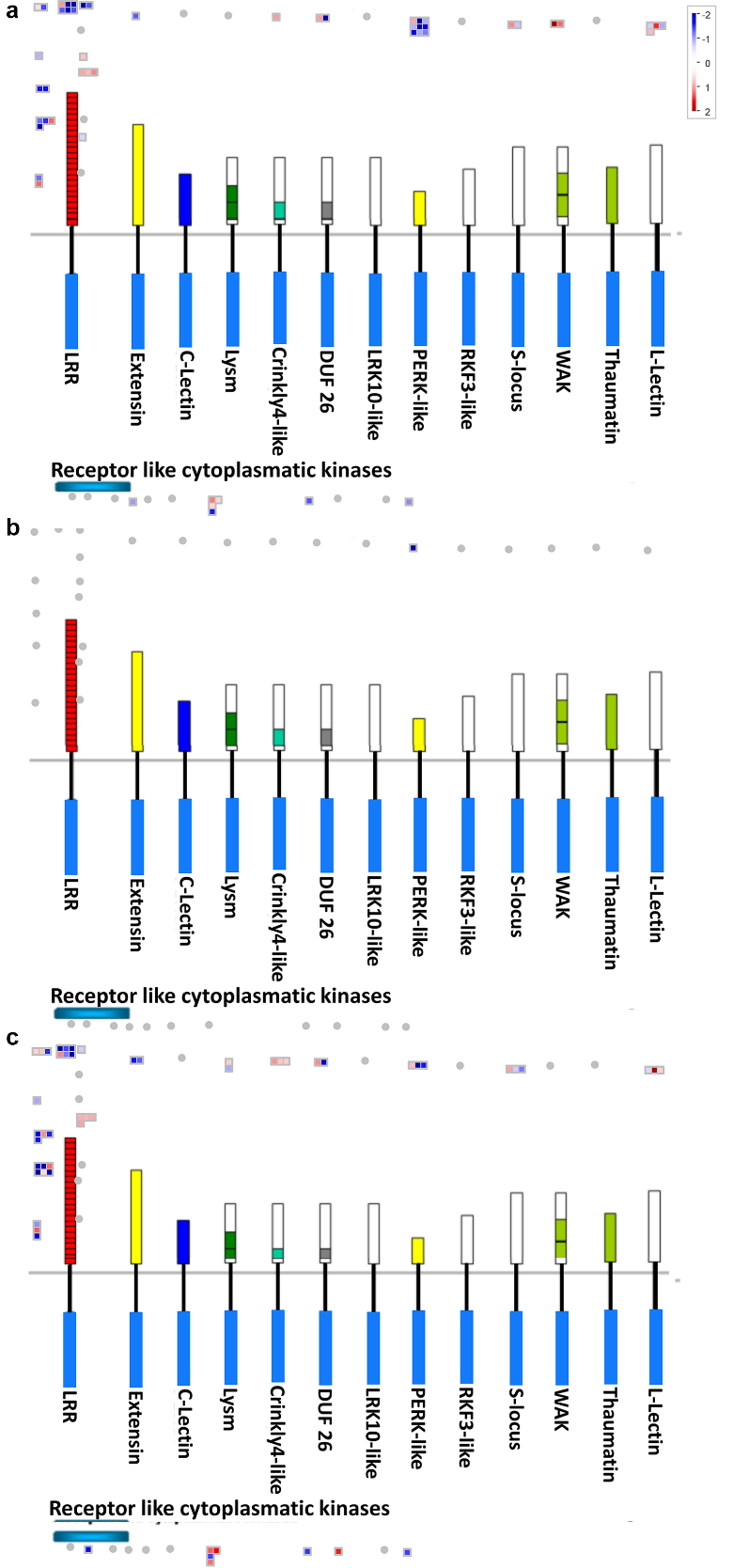


**Additional file 2: Figure S8.** Receptor-like kinases gene analyses of DEGs in ‘Brazilian’during early infection with both of Foc races*.* Analysis of the receptor-like kinases pathways of DEGs were performed using MapMan software. Red boxes mean up-regulated genes and green mean down-regulated genes. (a) Foc1 vs. CK;(b) Foc4 vs. CK; (c) Foc1 vs. Foc4.


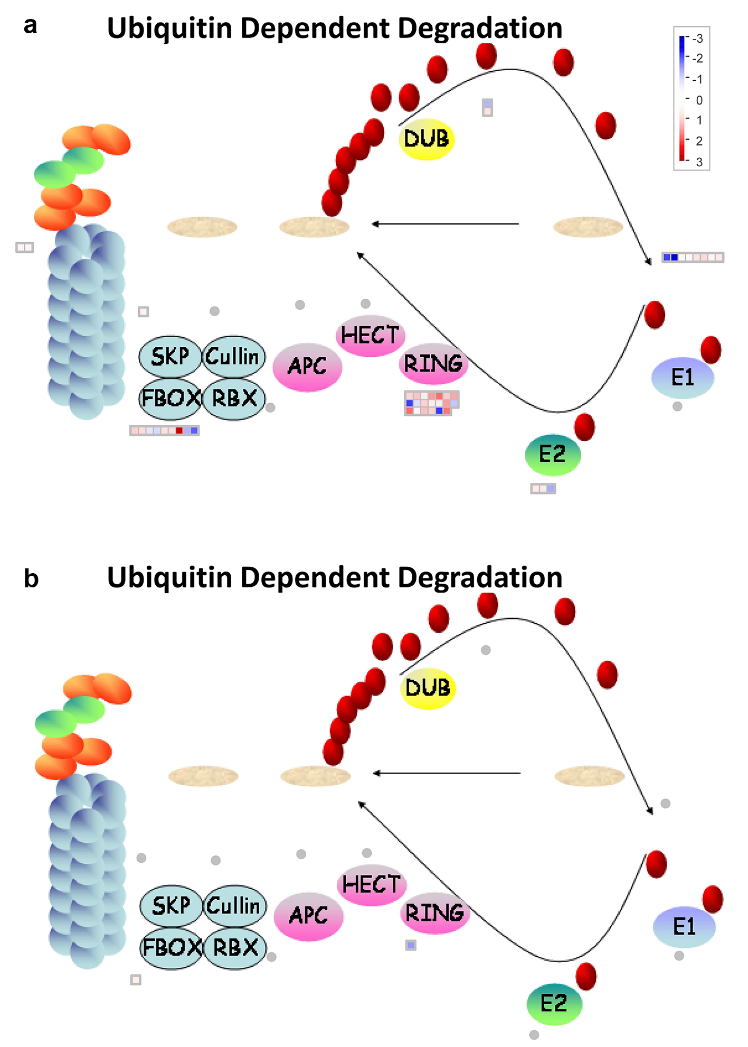


**Additional file 2: Figure S9.** Ubiquitin pathway analyses of DEGs in ‘Brazilian’during early infection with both of Foc races. Analysis of the ubiquitin pathway of DEGs was performed using MapMan software. Red boxes mean up-regulated genes and green mean down-regulated genes. (a) Foc1 vs. CK;(b) Foc4 vs. CK.


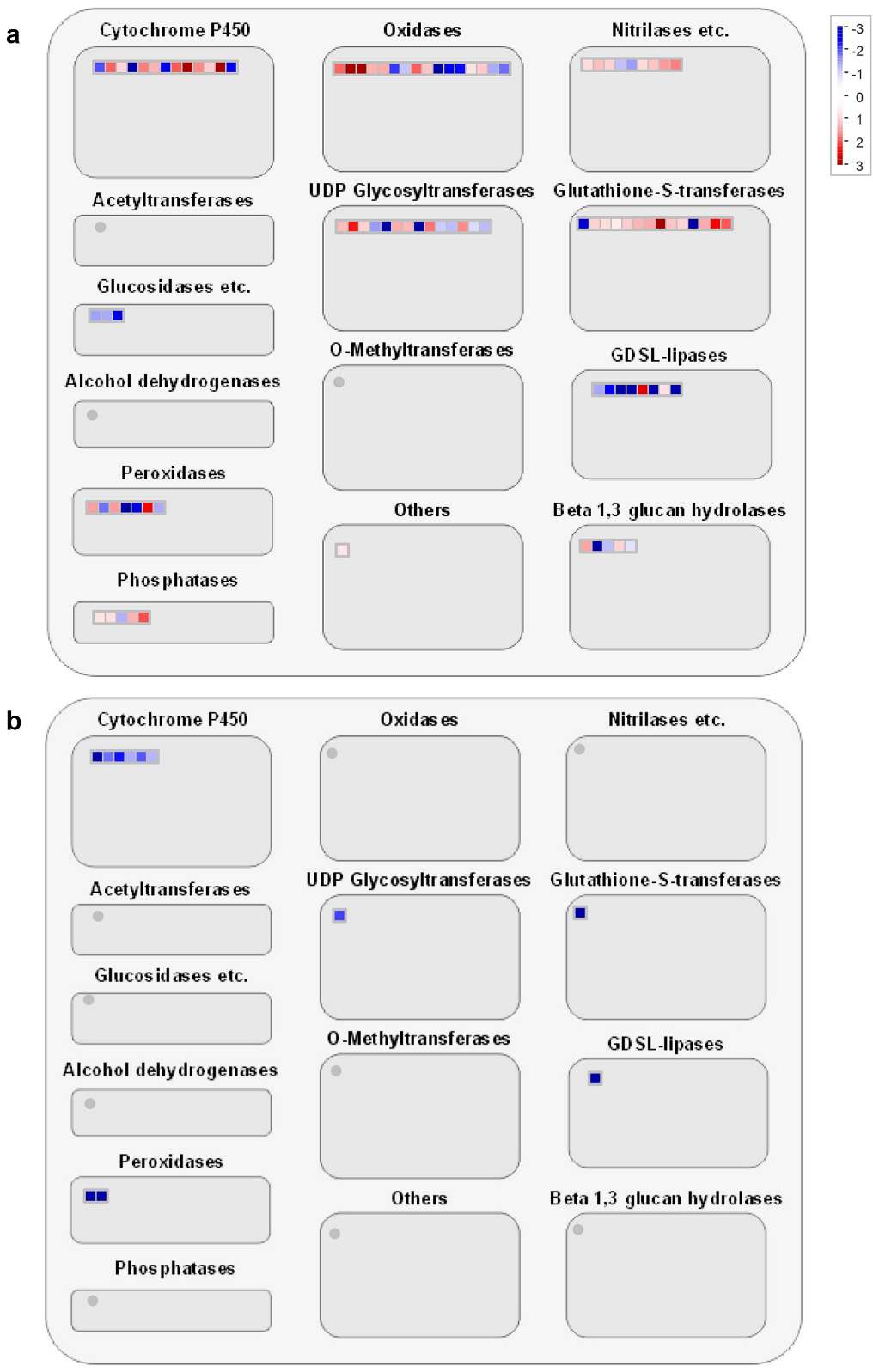


**Additional file 2: Figure S10.** Large enzyme families analyses of DEGs in ‘Brazilian’during early infection with both of Foc races. Analysis of the ubiquitin pathway of DEGs was performed using MapMan software. Red boxes mean up-regulated genes and green mean down-regulated genes. (a) Foc1 vs. CK;(b) Foc4 vs. CK.
